# A MicroRNA-Mediated Insulin Signaling Pathway Regulates the Toxicity of Multi-Walled Carbon Nanotubes in Nematode *Caenorhabditis elegans*

**DOI:** 10.1038/srep23234

**Published:** 2016-03-17

**Authors:** Yunli Zhao, Junnian Yang, Dayong Wang

**Affiliations:** 1 Key Laboratory of Environmental Medicine Engineering in Ministry of Education, Medical School, Southeast University, Nanjing 210009, China; 2 College of Life Sciences and Engineering, Chongqing Three Gorges University, Wanzhou 404000, China

## Abstract

The underlying mechanisms for functions of microRNAs (miRNAs) in regulating toxicity of nanomaterials are largely unclear. Using Illumina HiSeq^TM^ 2000 sequencing technique, we obtained the dysregulated mRNA profiling in multi-walled carbon nanotubes (MWCNTs) exposed nematodes. Some dysregulated genes encode insulin signaling pathway. Genetic experiments confirmed the functions of these dysregulated genes in regulating MWCNTs toxicity. In the insulin signaling pathway, DAF-2/insulin receptor regulated MWCNTs toxicity by suppressing function of DAF-16/FOXO transcription factor. Moreover, we raised a miRNAs-mRNAs network involved in the control of MWCNTs toxicity. In this network, *mir-355* might regulate MWCNTs toxicity by inhibiting functions of its targeted gene of *daf-2*, suggesting that *mir-355* may regulate functions of the entire insulin signaling pathway by acting as an upregulator of DAF-2, the initiator of insulin signaling pathway, in MWCNTs exposed nematodes. Our results provides highlight on understanding the crucial role of miRNAs in regulating toxicity of nanomaterials in organisms.

Carbon nanotubes (CNTs), an important class of engineered nanomaterials (ENMs), have numerous useful physicochemical properties. With the increase in CNTs manufacture, it is likely that an increasing exposure of CNTs to human and environmental organisms will occur^1^,^2^. Previous *in vitro* and *in vivo* studies have shown that CNTs exposure can cause several aspects of toxic effects on organisms through the induction of oxidative stress and/or inflammation^3^,^4^. The inhaled or instilled CNTs can be translocated into the secondary targeted organs such as central nervous system (CNS) through the blood in mammals^5^. Multi-walled carbon nanotubes (MWCNTs) are one important class of CNTs. MWCNTs are CNTs consisting of single-walled CNTs stacked one inside the other.

Previous studies have demonstrated the toxic effects of MWCNTs in inducing oxidative stress, altered immune or inflammatory response, reproductive toxicity, pulmonary toxicity, hepatotoxicology, and/or formation of mesothelioma in mammals^4^,^6^,^7^,^8^,^9^. Due to the properties of short lifespan, ease of manipulation, low cost, and well-described genetic and structural backgrounds, the model animal of *Caenorhabditis elegans* has been developed as an important non-mammalian alternative toxicity assay model^10^,^11^,^12^. Besides the lethal endpoint, a series of sublethal endpoints, such as intestinal reactive oxygen (ROS) production, locomotion behavior, and brood size, enable us be able to assess the toxic effects of environmental toxicants on the functions of both primary targeted organs such as intestine and secondary targeted organs such as neuron and reproductive organs^13^,^14^,^15^,^16^. *C. elegans* has been used in the studies of environmental safety evaluation, toxicological mechanism, and translocation of ENMs^12^. *C. elegans* has been successfully applied in the toxicological study of carbon-based ENMs such as CNTs and graphene oxide (GO)^17^,^18^,^19^,^20^,^21^,^22^. In *C. elegans*, MWCNTs exposure caused damage on the functions of both primary and secondary targeted organs^15^,^19^,^23^. Moreover, MWCNTs could be translocated into the secondary targeted organs such as reproductive organs in nematodes^15^,^19^.

To deeply elucidate the underlying molecular mechanism for nanotoxicity, the systems toxicology approach is a useful tool for nanotoxicological study^24^,^25^,^26^. microRNAs (miRNAs) are a class of short noncoding RNAs. miRNAs usually exhibit their biological functions by post-transcriptionally inhibiting the expression of targeted genes^27^. Based on SOLiD sequencing results, our previous study has identified the dysregulated miRNAs profiling induced by MWCNTs, and have confirmed the functions of some dysregulated miRNAs in regulating MWCNTs toxicity in nematodes^28^. However, the underlying mechanisms for the candidate miRNAs in regulating MWCNTs toxicity are still largely unclear. In this study, we first investigated the global mRNAs expression patterns in MWCNTs exposed nematodes using HiSeq 2000 sequencing technique. Again, with the aid of the identified miRNAs-mRNAs network involved in the control of MWCNTs toxicity, we examined the underling mechanism for *mir-355* in regulating MWCNTs toxicity through influencing the functions of insulin signaling pathways in nematodes. The raised miRNAs-mRNAs network involved in the control of MWCNTs toxicity and the identified *mir-355-*mediated insulin signaling pathway will highlight our understanding the underlying molecular mechanism of MWCNTs toxicity in organisms.

## Results

### Sequence assembly

Using the Illumina HiSeq^TM^ 2000 sequencing technique, transcriptomes from control and MWCNTs exposure groups were sequenced to obtain the clean reads data after filtering raw sequence data containing the adaptor fragments. Total reads of transcripts for the control group was 22159711, and total reads of transcripts for the MWCNTs exposure group was 32781156. We further found that 99.11% of the total reads of transcripts for control group and 99.15% of the total reads of transcripts for MWCNTs exposure group could be mapped among these reads. Number of the mapped bases for control group was 1130145261, and number of the mapped bases for MWCNTs exposure group was 1671838956.

Length distribution of sequences of protein coding region prediction (CDS) was shown in Fig. S1a. Approximately 21.97% of CDS was less than 1000 bp in length, 23.35% of CDS was between 1000 and 2000 bp in length, 13.54% of CDS was between 2000 and 3000 bp in length, 11.56% of CDS was between 3000 and 4000 bp in length, 7.8% of CDS was between 4000 and 5000 bp in length, 5.65% of CDS was between 5000 and 6000 bp in length, 4.08% of CDS was between 6000 and 7000 bp in length, and 12.03% of CDS was more than 7000 bp in length (Fig. S1a).

Distribution of CDS on chromosomes was shown in Fig. S1b. Approximately 20.14% of CDS was distributed on chromosome I, 22.4% of CDS was distributed on chromosome II, 18.55% of CDS was distributed on chromosome III, 21.61% of CDS was distributed on chromosome IV, 27.07% of CDS was distributed on chromosome V, and 17.87% of CDS was distributed on chromosome X (Fig. S1b).

### Transcriptomic changes induced by MWCNTs in nematodes

**Figure 1:**
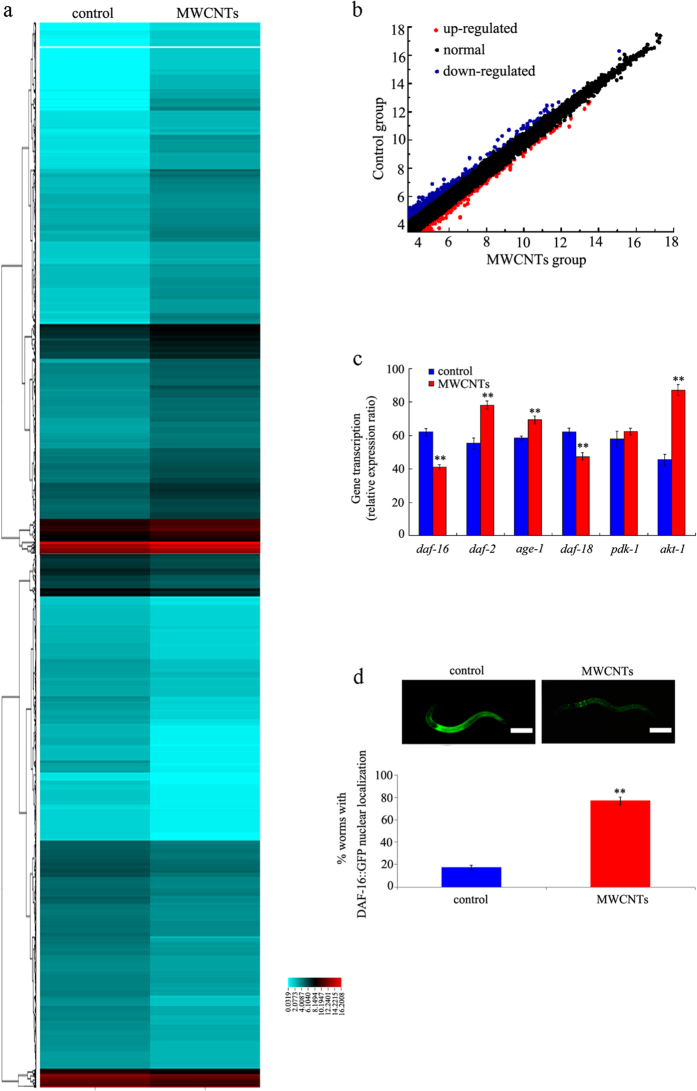
Dysregulated mRNAs induced by MWCNTs exposure. (**a**) Heatmap showing the expression of mRNAs obtained from control and MWCNTs-exposed nematodes. Relatively low expression levels are represented as blue, and relatively high expression levels are represented in red. (**b**) Scatter diagram of relationship between mRNA coverage of the control group and the MWCNTs exposure group. (**c**) qRT-PCR analysis of the expression expressions of some genes encoding insulin signaling pathway in nematodes exposed to MWCNTs. (**d**) MWCNTs exposure influenced the nuclear translocation of DAF-16::GFP in nematodes. Scale bar, 150 μm. MWCNTs (1 mg/L) exposure was performed from L1-larvae to young adult. Bars represent means ± S.E.M. ^**^P < 0.01 *vs* control.

We determined the dysregulated expression of mRNAs in MWCNTs exposed nematodes with the fold change analysis, which was further developed for the analysis based on statistical significance and use of a 2.0-fold change cutoff. We acquired annotations of differentially expressed genes by comparing the detected mRNA sequences with the databases of Genbank (Fig. 1a, Table S1). Transcriptional expression of 11849 genes was not significantly altered by MWCNTs exposure among the examined 13752 genes. That is, we totally identified 1903 differentially expressed mRNAs in MWCNTs exposed nematodes compared with control (Fig. 1b, Table S1). Among these 1903 candidate mRNAs, 924 mRNAs were up-regulated, and 993 mRNAs were down-regulated in MWCNTs exposed nematodes (Table S1). These results imply that the mRNA expression patterns may be globally influenced by MWCNTs exposure in nematodes.

Previous studies have suggested that MWCNTs toxicity formation may be at least due to the combinational effects of oxidative stress, and altered intestinal permeability in nematodes^15^,^19^. Pharmacological analysis suggested that treatment with NAC, an antioxidant, could effectively inhibit the induction of ROS production induced by MWCNTs exposure in nematodes (Fig. S2), suggesting the crucial role of oxidative stress in the induction of toxicity in nematodes exposed to MWCNTs. In the list of dysreguated mRNAs induced by MWCNTs exposure, we identified some genes associated with the control of oxidative stress, or intestinal development (Table S1). Based on the Illumina HiSeq^TM^ 2000 sequencing data, the dysregulated genes associated with the control of oxidative stress were *sod-2, sod-3, mev-1, isp-1, gas-1*, and *clk-1*, and the dysregulated genes associated with the control of intestinal development were *pgp-3, gem-4, par-3, pkc-3, ajm-1, lin-7, inx-3*, and *abts-4* (Table S1). Dysregulation of genes associated with the control of oxidative stress and intestinal development has been confirmed by our previous studies based on the qRT-PCR assay^15^,^19^.

### Confirmation of the dysregulated genes encoding insulin signaling pathway in MWCNTs exposed nematodes

In *C. elegans*, insulin signaling pathway is at least involved in the control of longevity, and stress response^29^. Among the MWCNTs-induced dysregulated genes encoding the insulin signaling pathway, we found that the transcriptional expressions of *daf-16*, and *daf-18* genes were decreased, and the transcriptional expressions of *age-1, daf-2, pdk-1*, and *akt-1* genes were increased in MWCNTs (1 mg/L) exposed nematodes compared with control (Table S1). Using the qRT-PCR technique, we further confirmed that the expression levels of *daf-16*, and *daf-18* genes were significantly decreased, and the expression levels of *age-1, daf-2*, and *akt-1* genes were significantly increased in MWCNTs (1 mg/L) exposed nematodes (Fig. 1c). In *C. elegans, daf-2* gene encodes a protein that is homologous to human insulin receptor InR, *age-1* gene encodes a protein that is homologous to human phosphoinositide 3-kinase PI3K, *daf-18* gene encodes a protein that is homologous to human lipid phosphatase PTEN, *akt-1* gene encodes a protein that is homologous to human serine/threonine kinase Akt/PKB, and *daf-16* gene encodes a protein that is homologous to human transcription factor FOXO.

Considering the fact that the insulin signaling pathway regulates biological processes such as longevity by limiting DAF-16 nuclear localization in nematodes^29^, we also investigated the effect of MWCNTs exposure on DAF-16 nuclear localization. After exposure to MWCNTs (1 mg/L), the percentage of nematodes with DAF-16::GFP in nucleus was significantly increased compared with control (Fig. 1d). Therefore, MWCNTs exposure may influence both the transcriptional activities of genes encoding insulin signaling pathway and the nuclear–cytoplasm translocation of DAF-16 in nematodes.

### Biological processes mediated by dysregulated genes in MWCNTs exposed nematodes

**Figure 2:**
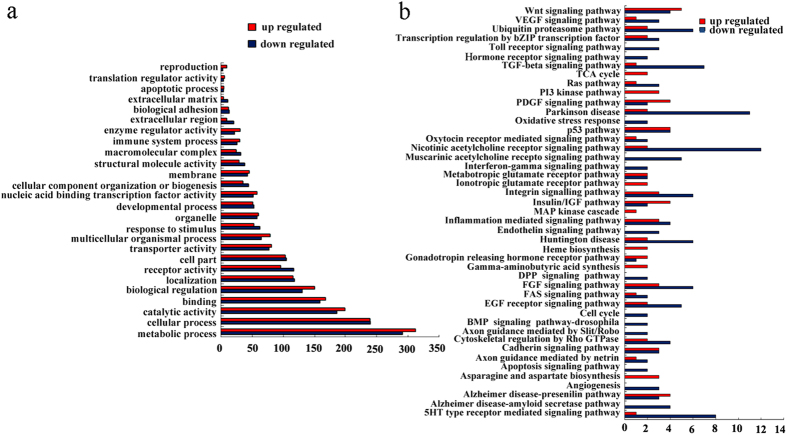
Analysis of gene ontology terms and signaling pathways. (**a**) Gene ontology terms with gene counts based on both down- and up-regulated mRNAs in MWCNTs-exposed nematodes. (**b**) Predicted KEGG signal pathways based on both down- and up-regulated mRNAs in MWCNTs-exposed nematodes.

Gene ontology analysis will provide ontology of the defined terms and the gene product properties in terms of their associated biological processes, cellular components, or molecular functions^30^. Based on the obtained dysregulated mRNAs, biological processes involved in the control of *in vivo* MWCNTs toxicity were first analyzed based on the gene ontology terms (Table S2 and Table S3). The significantly influenced gene ontology terms could be mainly classified into several categories, and these categories were at least associated with the biological processes of development, reproduction, cell adhesion, apoptosis, enzyme activity, cellular component, cellular localization and transportation, response to stimulus, immune response, cell metabolism, macromolecular complex, transcription, and translation in organisms (Fig. 2a).

KEGG pathway mapping is a bioinformatics resource used to map molecular data sets in genomics^31^, and the related signaling pathways can be further extracted by the pathway mining tool. Moreover, we employed KEGG pathway database to identify the signaling pathways mediated by the identified dysregulated mRNAs induced by MWCNTs exposure. We identified 38 signaling pathways for the down-regulated mRNAs and 36 signaling pathways for the up-regulated miRNAs in MWCNTs exposed nematodes (Table S4 and Table S5). The identified 38 signaling pathways contained the insulin signaling pathway (Fig. 2b, Table S4 and Table S5). The influenced signaling pathways by MWCNTs exposure mainly contained signaling pathways related to the development, cell cycle, cell death, oxidative stress response, cellular component, immune response, neuronal development and neurodegeneration, and cell metabolism (Fig. 2b). These results will be helpful for understanding and further elucidating the potential roles or functions of dysregulated genes in the formation of MWCNTs toxicity in nematodes.

### Genes encoding insulin signaling pathway were involved in the control of MWCNTs toxicity

**Figure 3:**
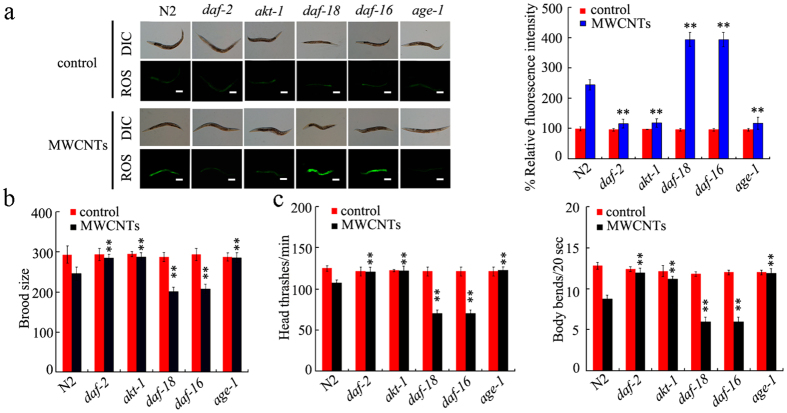
Genes encoding insulin signaling pathway were involved in the control of MWCNTs toxicity in nematodes. (**a**) Intestinal ROS production assay in mutants for genes encoding insulin signaling pathway. Scale bar, 150 μm. (**b**) Brood size assay in mutants for genes encoding insulin signaling pathway. (**c**) Locomotion behavior assay in mutants for genes encoding insulin signaling pathway. Locomotion behavior was assessed by the endpoints of head thrash and body bend. The used nematode strains were wild-type N2, *daf-16(mu86), daf-2(e1370), age-1(hx546), daf-18(e1375)*, and *akt-1(ok525)*. MWCNTs (1 mg/L) exposure was performed from L1-larvae to young adult. Bars represent means ± S.E.M. ^**^P < 0.01 *vs* N2.

Among the candidate genes encoding insulin signaling pathway dysregulated by MWCNTs, mutation of *daf-2, age-1*, or *akt-1* gene led to the obvious inhibition in induction of ROS production in intestine and the significant increase in brood size or locomotion behavior in MWCNTs (1 mg/L)-exposed nematodes (Fig. 3). In contrast, mutation of *daf-16* or *daf-18* gene resulted in the enhanced induction of ROS production in intestine and the more severe decrease in brood size or locomotion behavior in MWCNTs (1 mg/L)-exposed nematodes (Fig. 3). These results demonstrate that the insulin signaling pathway may play a crucial role in regulating the MWCNTs toxicity in nematodes.

### A miRNAs-mRNAs network involved in the control of MWCNTs toxicity

In *C. elegans*, it has been shown that MWCNTs exposure could induce the dysregulation of a series of miRNAs^28^. Among the dysregulated miRNAs, the further bioinformatics analysis demonstrated that *lin-4, mir-228, mir-249, mir-47, mir-355, mir-45, mir-2210, mir-57, mir-1018, mir-360, mir-64, mir-2209, mit-793, mir-1830, mir-2210, mir-83, mir-789*, and *mir-806* might be involved in the control of MWCNTs toxicity through affecting the functions of identified dysregulated genes in exposed nematodes (Table S6). Moreover, we found that the *isp-1* gene might serve as a molecular target for *mir-249* to regulate the induction of oxidative stress in MWCNTs exposed nematodes (Table S6). Mutation of *mir-249* enhanced the induction of ROS production in MWCNTs-exposed nematodes; however, mutation of *isp-1* inhibited the induction of ROS production in MWCNTs-exposed nematodes (Fig. S3). Moreover, mutation of *isp-1* gene suppressed the enhanced induction of ROS production observed in MWCNTs-exposed *mir-249(n4983)* mutants (Fig. S3). The *ajm-1* gene might serve as a molecular target for *mir-64* to regulate the intestinal function in MWCNTs exposed nematodes (Table S6). The relative fluorescence intensity of Nile Red signals in the intestine can be used to reflect the state of intestinal permeability in nematodes^15^,^22^. Mutation of *mir-64* exhibited the similar Nile Red staining pattern to that of wild-type nematodes; however, mutation of *ajm-1* induced a significant increase in relative fluorescence intensity of Nile Red signals in the intestine of nematodes (Fig. S4). Moreover, mutation of *ajm-1* gene also induced a significant increase in relative fluorescence intensity of Nile Red signals in the intestine of in *mir-64(nDf52)* mutants (Fig. S4). Because Nile Red can also be used to label fat storage, we further investigated the triglyceride amount. There was no significant difference among the *ajm-1(RNAi), mir-64(nDf52), mir-64(nDf52);ajm-1(RNAi)*, and wild-type nematodes (data not shown). These results imply that a specific miRNAs-mRNAs network may play a key role in regulating the MWCNTs toxicity at least through influencing the induction of oxidative stress and intestinal development and function in nematodes.

### DAF-2 functioned as a molecular target for *mir-355* in regulating MWCNTs toxicity in nematodes

**Figure 4:**
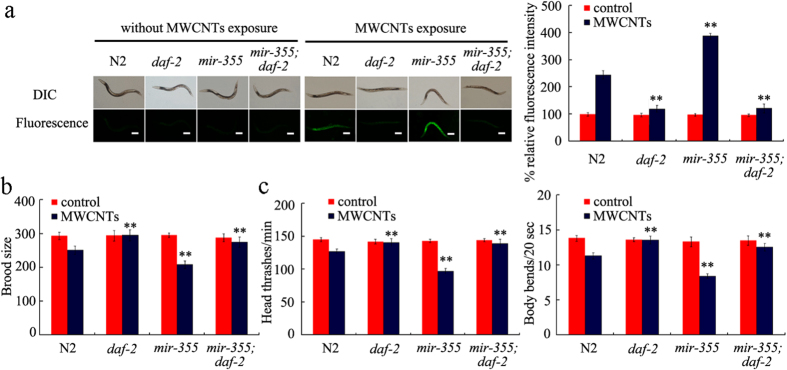
Genetic interaction of *mir-355* with DAF-2 in regulating MWCNTs toxicity in nematodes. (**a**) Genetic interaction of *mir-355* with DAF-2 in regulating MWCNTs toxicity on the induction of intestinal ROS production in nematodes. Scale bar, 150 μm. (**b**) Genetic interaction of *mir-355* with DAF-2 in regulating MWCNTs toxicity on the reduction in brood size in nematodes. (**c**) Genetic interaction of *mir-355* with DAF-2 in regulating MWCNTs toxicity on the decrease in locomotion behavior in nematodes. Locomotion behavior was assessed by the endpoints of head thrash and body bend. The used nematode strains were wild-type N2, *mir-355(n4618), daf-2(RNAi)*, and *mir-355(n4618);daf-2(RNAi)*. MWCNTs (1 mg/L) exposure was performed from L1-larvae to young adult. Bars represent means ± S.E.M. ^**^P < 0.01 *vs* N2.

The bioinformatics analysis also suggested that *daf-2* gene might serve as a molecular target for *mir-355* in regulating the MWCNTs toxicity in nematodes. Our previous study has suggested that mutation of *mir-355* resulted in the susceptible property of nematodes to MWCNTs toxicity with the aid of brood size, head thrash, body bend, intestinal autofluorescence, and intestinal ROS production as the toxicity assessment endpoints^28^. Assuming that *daf-2* is the potential targeted gene for *mir-355*, the double mutant of *mir-355;daf-2* should exhibit the similar phenotypes to those in *daf-2* mutant. As shown in Fig. 4, the double mutant of *mir-355(n4618);daf-2(RNAi)* exposed to MWCNTs (1 mg/L) showed the similar phenotypes of induction of intestinal ROS production, brood size, and locomotion behavior to those in *daf-2(RNAi)* nematodes exposed to MWCNTs (1 mg/L). These genetic evidences have demonstrated that *daf-2* gene may be the potential targeted gene for *mir-355* in regulating the MWCNTs toxicity in nematodes.

### Genetic interaction of DAF-2 with DAF-16 in regulating MWCNTs toxicity in nematodes

**Figure 5:**
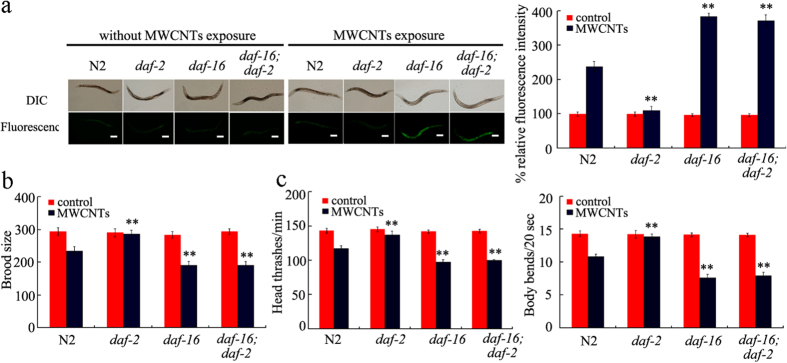
Genetic interaction of DAF-2 with DAF-16 in regulating MWCNTs toxicity in nematodes. (**a**) Genetic interaction of DAF-2 with DAF-16 in regulating MWCNTs toxicity on the induction of intestinal ROS production in nematodes. Scale bar, 150 μm. (**b**) Genetic interaction of DAF-2 with DAF-16 in regulating MWCNTs toxicity on the reduction in brood size in nematodes. (**c**) Genetic interaction of DAF-2 with DAF-16 in regulating MWCNTs toxicity on the decrease in locomotion behavior in nematodes. Locomotion behavior was assessed by the endpoints of head thrash and body bend. The used nematode strains were wild-type N2, *daf-16(mu86), daf-2(e1370)*, and *daf-16(mu86);daf-2(e1370)*. MWCNTs (1 mg/L) exposure was performed from L1-larvae to young adult. Bars represent means ± S.E.M. ^**^P < 0.01 *vs* N2.

In *C. elegans*, DAF-2 functions upstream of DAF-16 to regulate some phenotypes of animals such as longevity^29^. In the present study, we further observed that the double mutant of *daf-16(mu86);daf-2(e1370)* exposed to MWCNTs (1 mg/L) exhibited the similar phenotypes of induction of intestinal ROS production, brood size, and locomotion behavior to those in *daf-16(mu86)* mutant exposed to MWCNTs (1 g/L) (Fig. 5). That is, after exposure to MWCNTs (1 mg/L), mutation of *daf-16* gene suppressed the phenotypes of induction of intestinal ROS production, brood size, and locomotion behavior in *daf-2(e1370)* mutants (Fig. 5). These results suggest that DAF-2 may function upstream of DAF-16 to regulate the MWCNTs toxicity in nematodes.

### Effects of *ins-7* overexpression on the MWCNTs toxicity in nematodes

**Figure 6:**
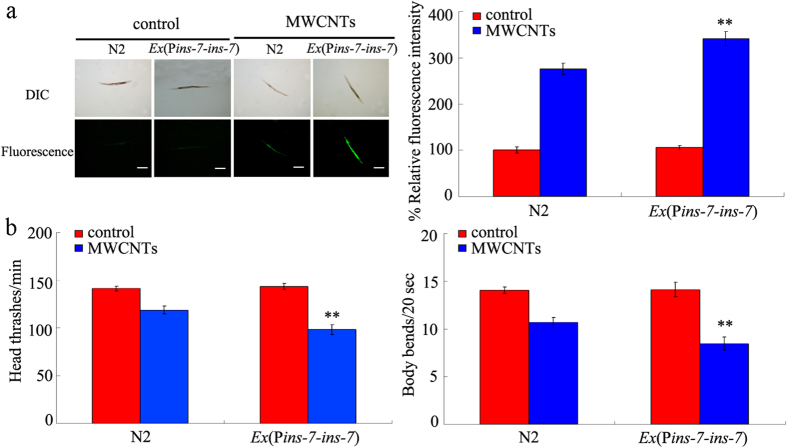
Effects of *ins-7* overexpression on the MWCNTs toxicity in nematodes. (**a**) Effects of *ins-7* overexpression on the MWCNTs toxicity in inducing ROS production in nematodes. Scale bar, 150 μm. (**b**) Effects of *ins-7* overexpression on the MWCNTs toxicity in decreasing locomotion behavior in nematodes. Locomotion behavior was assessed by the endpoints of head thrash and body bend. MWCNTs (1 mg/L) exposure was performed from L1-larvae to young adult. Bars represent means ± S.E.M. ^**^P < 0.01 *vs* N2.

In *C. elegans, ins-7* gene encodes an insulin peptide, which acts as a ligand for the insulin receptor of DAF-2. Overexpression of *ins-7* gene did not induce the significant induction of ROS production or obviously affect the locomotion behavior compared with wild-type nematodes (Fig. 6). However, after MWCNTs exposure, we found that overexpression of *ins-7* gene induced a more pronounced induction of ROS production and decrease in locomotion behavior than wild-type nematodes (Fig. 6). These results suggest that overexpression of *ins-7* may induce a susceptible property of nematodes to MWCNTs toxicity in nematodes. In addition, our data imply that INS-7 may act as an agonist for DAF-2 in the control of MWCNTs toxicity in nematodes.

## Discussion

In the present study, we first performed the Illumina HiSeq^TM^ 2000 sequencing, and obtained the dysregulated mRNA expression profiling in MWCNTs exposed nematodes. Among the identified dysregulated genes based on the Illumina HiSeq^TM^ 2000 sequencing data, we found that some genes associated with the control of oxidative stress or intestinal development have been identified previously in MWCNTs exposed nematodes based on qRT-PCR assay^15^,^19^. Moreover, both the gene ontology terms assay and the KEGG signaling pathway assay have suggested the influences of MWCNTs exposure on biological processes of development, reproduction, cellular component, oxidative stress response, immune response, and cell metabolism (Fig. 2a,b). First of all, these results suggest the potential cellular, biochemical, and molecular mechanisms for MWCNTs toxicity in nematodes. Moreover, these results also imply some previously unknown adverse effects of MWCNTs in nematodes, such as the induction of abnormal neuronal development and neurodegeneration (Fig. 2b).

Among the dysregulated genes based on Illumina HiSeq^TM^ 2000 sequencing data, interestingly, we found that some genes encode the insulin signaling pathway in nematodes. The qRT-PCR assay confirmed the expression patterns of some genes encoding the insulin signaling pathway (Fig. 1c). In addition, like the effects of some other toxicants, MWCNTs exposure increased the percentage of cells with DAF-16::GFP nuclear localization (Fig. 1d). During the molecular control of arsenite or PM_2.5_ toxicity by insulin signaling pathway, the DAF-16:: GFP nuclear localization was also significantly increased^32^,^33^. Among the genes encoding insulin signaling pathway, genetic evidence has further proven the functions of *daf-2, age-1, akt-1, daf-18*, and *daf-16* genes in regulating MWCNTs toxicity in nematodes (Fig. 3). Mutation of *daf-2, age-1, akt-1, daf-18*, or *daf-16* gene altered the phenotypes of induction of intestinal ROS production, brood size, and locomotion behavior in MWCNTs exposed nematodes (Fig. 3). Moreover, genetic experiments have indicated that DAF-2/insulin receptor could regulate the MWCNTs toxicity by suppressing the function of DAF-16/FOXO transcription factor in nematodes (Fig. 5), suggesting the genetic interaction of components in the insulin signaling pathway in regulating MWCNTs toxicity. Interestingly, besides the insulin signaling pathway, many other important signaling pathways such as Wnt signaling pathway, Toll receptor signaling pathway, TGF-beta signaling pathway, MAPK signaling pathway, and cadherin signaling pathway might also be involved in the control of MWCNTs toxicity based on the bioinformatics analysis (Fig. 3b).

Moreover, in this study, we raised a miRNAs-mRNAs network involved in the control of MWCNTs toxicity based on the obtained dysregulated mRNA profiling and the previously identified dysregulated miRNA profiling in MWCNTs exposed nematodes^28^. This miRNAs-mRNAs network provides the important clues for the further elucidation of underlying mechanisms for the candidate miRNAs in regulating the MWCNTs toxicity in nematodes. In this miRNAs-mRNAs network, we found that the candidate miRNAs might function through some important signaling pathways to regulate the MWCNTs toxicity in nematodes (Table S6). These important signaling pathways at least contained the insulin signaling pathway, p38 MAPK signaling pathway, oxidative stress signaling pathway, and JNK signaling pathway (Table S6).

**Figure 7:**
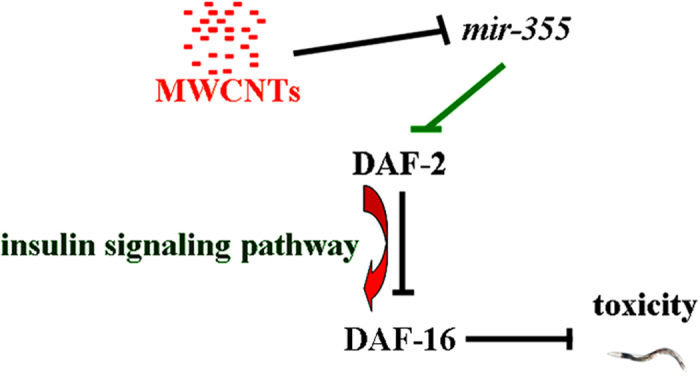
A diagram showing the *mir-355* mediated signaling pathway in the control of MWCNTs toxicity in nematodes.

In this miRNAs-mRNAs network, more interestingly, we found that *daf-2* gene may act as the potential targeted gene for *mir-355* in regulating the MWCNTs toxicity (Figs 4 and 7, Table S6). The genetic experiments have demonstrated that *mir-355* could regulate the MWCNTs toxicity by suppressing the functions of *daf-2* gene in nematodes (Fig. 4). In cells of nematodes, DAF-2 is the initiator of insulin signaling pathway. That is, *mir-355* may regulate the functions of entire insulin signaling pathway by acting as an upregulator of DAF-2 in MWCNTs-exposed nematodes. This elucidated molecular mechanism for the function of *mir-355* in regulating the MWCNTs toxicity provides an important highlight on the understanding the roles of miRNA molecules in regulating biological behavior of ENMs in organisms.

In the present study, our data suggest the important role of insulin signaling pathway in the control of MWCNTs toxicity in nematodes (Fig. 7). In the insulin signaling pathway, the FOXO transcriptional factor of DAF-16 acts through its targets such as the proteins of Mn-SODs to exert its effects on nematodes^29^. Previous study has suggested that MWCNTs exposure could dysregulate the expression of *sod-2* and *sod-3* genes encoding Mn-SODs^19^. Therefore, DAF-16 can potentially at least regulate the MWCNTs toxicity through affecting the functions of its targeted Mn-SOD proteins.

In conclusion, using the Illumina HiSeq^TM^ 2000 sequencing technique, we first obtained the dysregulated mRNA profiling in MWCNTs-exposed nematodes. The bioinformatics analysis has implied the biological processes mediated by these dysregulated genes in MWCNTs-exposed nematodes. Among the dysregulated genes induced by MWCNTs exposure, some genes encode the insulin signaling pathway, and genetic analysis confirmed their functions in regulating the MWCNTs toxicity. Moreover, we raised a miRNAs-mRNAs network involved in the control of MWCNTs toxicity in nematodes. In this miRNAs-mRNAs network, we provide the evidence to prove the regulation relationship between *mir-355* and DAF-2/insulin receptor in regulating the MWCNTs toxicity in nematodes. Considering the key and conserved functions of insulin signaling pathway in the control of many biological processes in different organisms, our work on the function of *mir-355*-mediated insulin signaling pathway in the control of MWCNTs toxicity will strengthen our understanding the crucial roles and the underlying mechanism of miRNAs in the control of biological behavior of ENMs in organisms.

## Methods

### Chemicals

The used MWCNTs (diameter: 10–20 nm, length: 5–15 mm) were from Shenzhen Nanotech. Port Co. Ltd (Shenzhen, China). Morphology of MWCNTs in K-medium was examined by transmission electron microscopy (TEM, JEM-200CX, JEOL, Japan) (Fig. S5). Zeta potential of MWCNTs was analyzed by Nano Zetasizer (Nano ZS90, Malvern Instrument, UK), and the zeta potential of MWCNTs in K-medium was −32.9 ± 2.1 mV.

MWCNTs were dispersed in K medium to prepare the stock solution (1 mg/mL). And then, MWCNTs were sonicated for 30 min (40 kHz, 100 W), and diluted to the used concentration (1 mg/L) with K medium just prior to exposure.

### *C. elegans* maintenance

The used nematodes were wild-type N2, mutants of *daf-16(mu86)*^34^, *daf-2(e1370)*^35^, *age-1(hx546)*^36^, *daf-18(e1375)*^37^, *akt-1(ok525)*^38^, *isp-1(qm150)*^39^, *mir-355(n4618)*^40^, *mir-64(nDf52)*^40^, *mir-249(n4983)*^40^, *mir-355(n4618);daf-2(e1370), daf-16(mu86);daf-2(e1370)*, and *isp-1(qm150);mir-249(n4983)* and transgenic strains of *Ex*(P*ins-7-ins-7*) and *zIs356*[P*daf-16::daf-16a/b::GFP* + *rol-6*], which were maintained on nematode growth medium (NGM) plates seeded with *Escherichia coli* OP50 at 20 °C as described^10^. All the used single mutants are loss-of-function mutants. Some of the used *C. elegans* strains were originally obtained from *Caenorhabditis* Genetics Center (CGC, funded by NIH Office of Research Infrastructure Programs (P40 OD010440)). Gravid nematodes collected from NGM plates were transferred into centrifuge tubes to be lysed with a bleaching mixture (0.45 M NaOH, 2% HOCl). Age synchronous populations of L1-larvae were obtained as described previously^41^.

### MWCNTs exposure

MWCNTs exposure was performed from L1-larvae to young adult (prolonged exposure) in 12-well sterile tissue culture plates at 20 °C in the presence of food of OP50. After MWCNTs exposure, nematodes were used for toxicity assessment with the aid of reproduction, locomotion behavior, and intestinal ROS production as endpoints.

### RNA-seq library preparation and HiSeq 2000 sequencing

Previous study has suggested that exposure to MWCNTs (1 mg/L) could cause the adverse effects on nematodes^19^. Total RNAs from wild-type N2 nematodes exposed to MWCNTs (1 mg/L) or without MWCNTs exposure were isolated using Trizol Reagent (Invitrogen, UK) according to the manufacturer’s instructions. The contaminating DNA was removed with Ambion TURBO DNA-free kit (Applied Biosystems, Austin, TX, USA). After determination of RNA quality using a Nano Photometer P-Class, mRNA libraries were constructed using RNA-seq Sample Preparation kit (Illumina, Inc., San Diego, CA, USA). Illumina HiSeq^TM^ 2000 sequencing plat form was applied to obtain 22.1 million and 20.7 million 100-nucleotide paired-end reads for control and MWCNTs exposure group mRNAs, respectively.

### RNA-seq data analysis

Quality of the reads was checked with Fast QC. Draft genome of *C. elegans* (version WS220, Release 62, Ensembl, fttp://ftp.ensembl.org/pub/release-62/fasta/caenorhabditis_elegans/dna/) was used as the reference-guided mapping of transcriptome sequencing reads with TopHat 1.3.1 that uses the Bowtie and SAMtools 0.1.16. Total read numbers of control group and MWCNTs exposure group data sets were normalized to equal levels. The relative gene abundance was defined by log10 of the normalized read number. Transcripts with false discovery rate-corrected *p*-values < 0.05 and fold change >2 were defined as differentially expressed.

### Pathway analysis

Differentially expressed genes (DEGs) were analyzed by default parameters to adjust the *p* value with the whole genome set as a background. Enriched gene ontology terms were generated using gene ontology database (http://www.Geneontology.org/) based on blast with *C. elegans* database, a reference background. Gene ontology analysis and enrichment were analyzed separately for upregulated and downregulated genes using DAVID Functional Annotation Tool. The Kyoto Encyclopedia of Genes and Genomes (KEGG) Orthology database (http://www.genome.jp/kegg/ko.html) was used for signaling pathway mapping analysis.

### Bioinformatics analysis for miRNAs-mRNAs network

TargetScan was useful for predicting biological targets of miRNAs by searching for the presence of conserved sites that match seed region of each miRNA. The corresponding miRNA(s) for dysregulated genes induced by MWCNTs were predicted by TargetScan version 6.2 (http://www.targetscan.org/worm_52/). We then searched whether the dysregulated genes were the possible biological targets for dysregulated miRNAs induced by MWCNTs exposure^28^. We first used the TargetScan to predict the targets of candidate miRNAs by searching for the presence of conserved sites that can match the seed region of each miRNA, and then validated the results using other algorithms including mirBase, PicTar, and miRanda.

### Reverse-transcription and quantitative real-time polymerase chain reaction (qRT-PCR)

Total RNA was isolated from nematodes using Trizol (Invitrogen, UK) according to manufacturer’s protocols. RNAs were first assessed for their purity and concentration by OD260/280 in a spectrophotometer, and then used for cDNA synthesis performed in a 12.5 μL reaction volume containing 625 ng total RNA, 0.5 mM reverse-transcript primers, 50 mM Tris-HCl, 75 mM KCl, 3 mM MgCl_2_, 10 mM dithiothreitol, 20 units ribonuclease inhibitor and 100 U reverse transcriptase (Takara, China). After cDNA synthesis, relative expression levels of examined genes were determined by real-time PCR in an ABI 7500 real-time PCR system with Evagreen (Biotium, USA). Relative quantification of targeted genes in comparison to reference *tba-1* gene encoding tubulin was determined. The final results were expressed as relative expression ratio between the targeted gene and the reference gene. Primer information was shown in Table S7. All reactions were performed in triplicate.

### Toxicity assessment

Reproduction was assessed by the endpoint of brood size. Method for reproduction assay was performed as described previously^42^,^43^. Number of offspring at all stages beyond the egg was counted. Twenty nematodes were examined per treatment, and three replicates were performed.

Locomotion behavior was assessed by the endpoints of head thrash and body bend. Methods for locomotion behavior assay were performed as described previously^44^,^45^. A head thrash was defined as a change in the direction of bending at the mid body. A body bend was counted as a change in the direction of the part of the nematodes corresponding to the posterior bulb of the pharynx along the *y* axis, assuming that nematode was traveling along the *x* axis. Twenty nematodes were examined per treatment, and three replicates were performed.

Method for ROS production assay was performed as described previously^46^,^47^. Nematodes were transferred to 1 μM of 5’,6’-chloromethyl–2’,7’dichlorodihydro-fluorescein diacetate (CM-H2DCFDA) in 12-well sterile tissue culture plates to incubate for 3 h at 20 °C. Nematodes were then mounted on 2% agar pads for the examination at 488 nm of excitation wavelength and 510 nm of emission filter with a laser scanning confocal microscope (Leica, TCS SP2, Bensheim, Germany). Relative fluorescence intensities in the intestines were semi-quantified. Thirty nematodes were examined per treatment, and three replicates were performed.

### RNA interference (RNAi)

To construct the double mutant of *mir-355(n4618);daf-2(RNAi)* or *mir-64(nDf52);ajm-1(RNAi)*, RNAi was performed by feeding nematodes with *E. coli* strain HT115 (DE3) expressing double-stranded RNA homologous to a target gene as described previously^48^,^49^. *E. coli* HT115 (DE3) grown in LB broth containing ampicillin (100 μg/mL) at 37 °C overnight was plated onto NGM plates containing ampicillin (100 μg/mL) and isopropyl 1-thio-β-D-galactopyranoside (IPTG, 5 mM) to induce the double-stranded RNA. RNAi-expressing bacteria were allowed to grow at 37 °C overnight. For strain feeding, about 50 synchronized L1 larvae were transferred to RNAi or vector control plates for 2 days at 20 °C until nematodes became gravid. Gravid adults were transferred to fresh RNAi-expressing bacterial lawns to lay eggs for 2-h so as to obtain the second generation RNAi population. Eggs were allowed to develop at 20 °C to L1 larvae for the subsequent exposure.

### Pharmacological assay

The MWCNTs (1 mg/L) exposure was performed from L1-larvae to young adult, and then nematodes were further treated with 5 mM of N-acetyl-L-cysteine (NAC) for 24 h^50^. NAC is an antioxidant potentially used to treat mitochondrial dysfunction, and treatment with 5 mM of NAC did not influence survival of nematodes^51^. Graphs are representative of five trials.

### Nile Red staining

Nile Red (Molecular Probes, Eugene, OR) was dissolved in acetone to generate a 0.5 mg/mL stock solution, and freshly diluted in 1× PBS buffer to 1 μg/mL. Approximately 150 mL of the diluted solution was poured onto NGM plates already seeded with OP50. Nematodes were cultured on the plates for 3 days before observation. Thirty nematodes were examined per treatment, and three replicates were performed.

### DNA constructs and germline transformation

The full fragment of *ins-7* gene was amplified by PCR from wild-type *C. elegans* genomic DNA, and then inserted into the pPD95_77 vector in the sense orientation. Germline transformation was performed by coinjecting the testing DNA at a concentration of 10–40 μg/mL and the marker DNA of *unc-119(+)* at a concentration of 60 μg/mL into the gonad of nematodes^52^.

### Statistical analysis

All data in this article were expressed as means ± standard error of the mean (S.E.M.). Graphs were generated using Microsoft Excel software (Microsoft Corp., Redmond, WA). Statistical analysis was performed using SPSS 12.0 software (SPSS Inc., Chicago, USA). Differences between groups were determined using analysis of variance (ANOVA). The probability levels of 0.05 and 0.01 were considered to be statistically significant.

## Additional Information

**How to cite this article**: Zhao, Y. *et al*. A MicroRNA-Mediated Insulin Signaling Pathway Regulates the Toxicity of Multi-Walled Carbon Nanotubes in Nematode *Caenorhabditis elegans. Sci. Rep.* **6**, 23234; doi: 10.1038/srep23234 (2016).

## Supplementary Material

Supplementary Information
