# Supplementary material for: A MicroRNA-Mediated Insulin Signaling Pathway Regulates the Toxicity of Multi-Walled Carbon Nanotubes in Nematode Caenorhabditis elegans
Source: Sci Rep. 2016 Mar 17;6:23234. doi: 10.1038/srep23234 (PMC4794644; doi:10.1038/srep23234)
Supplement: Supplementary Information [file srep23234-s1.doc]

**A MicroRNA-Mediated Insulin Signaling Pathway Regulated the Toxicity of Multi-Walled Carbon Nanotubes in Nematode *Caenorhabditis elegans***

Yunli Zhao^1 *^, Junnian Yang^1, 2, *^ & Dayong Wang^1, **^

^1^Key Laboratory of Environmental Medicine Engineering in Ministry of Education, Medical School, Southeast University, Nanjing 210009, China

^2^College of Life Sciences and Engineering, Chongqing Three Gorges University, Wanzhou 404000, China

^*^They contributed equally to this work.

^**^Correspondence and requests for materials should be addressed to D.W. (dayongw@seu.edu.cn)

**Supporting Information:**

**
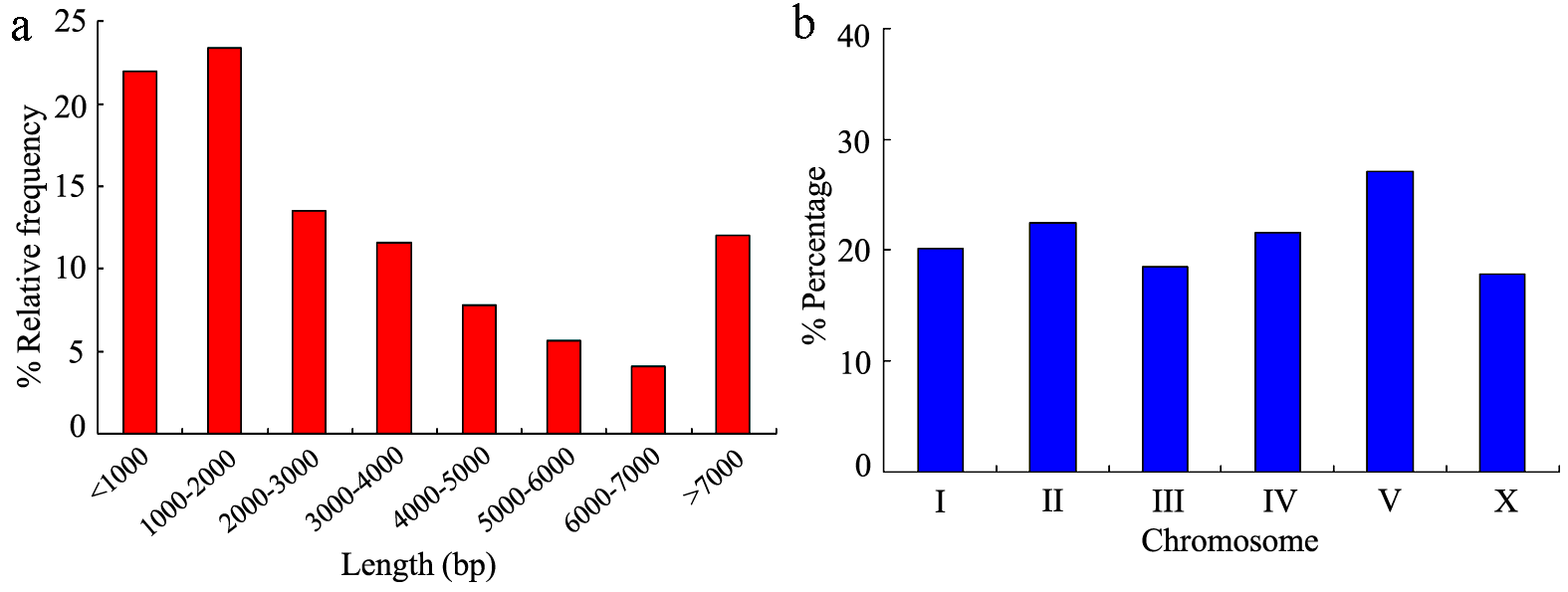
**

**Figure S1 | Properties of nucleotide sequences.**  (a) Length distribution of CDS extracted from annotated unigenes. (b) Distribution of CDS extracted from annotated unigenes on different chromosomes.

**
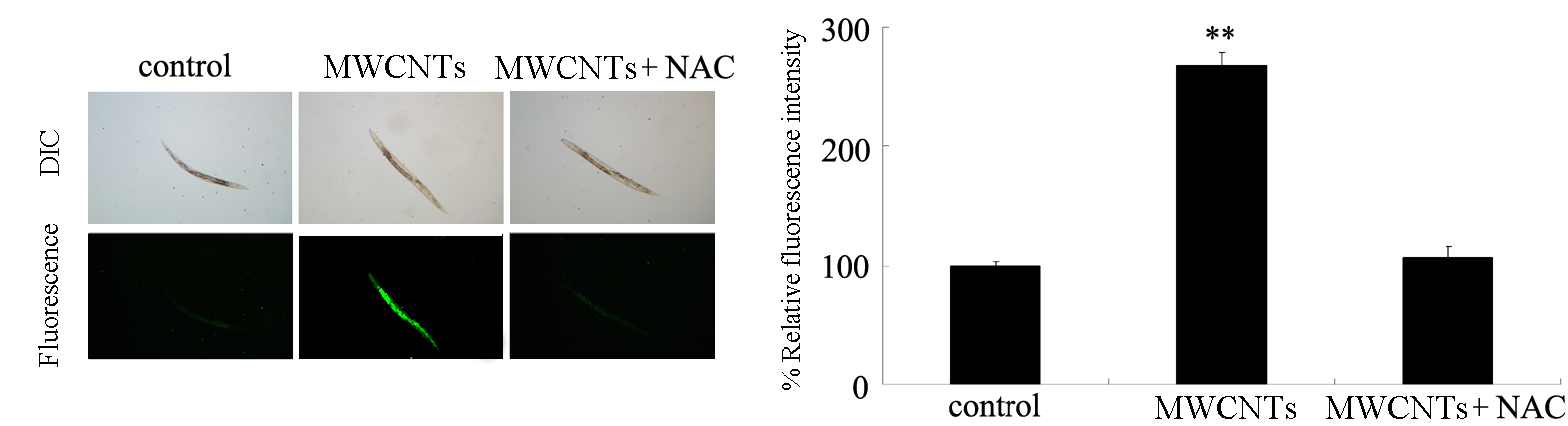
**

**Figure S2 | NAC treatment inhibited the induction of ROS production in nematodes exposed to MWCNTs.**  Nematodes were first exposed to MWCNTs (1 mg/L) from L1-larvae to young adult, and then treated with 5mM of NAC for 24 h. NAC, N-acetyl-L-cysteine. Bars represent means ± S.E.M. ^**^*P* < 0.01 *vs* control.


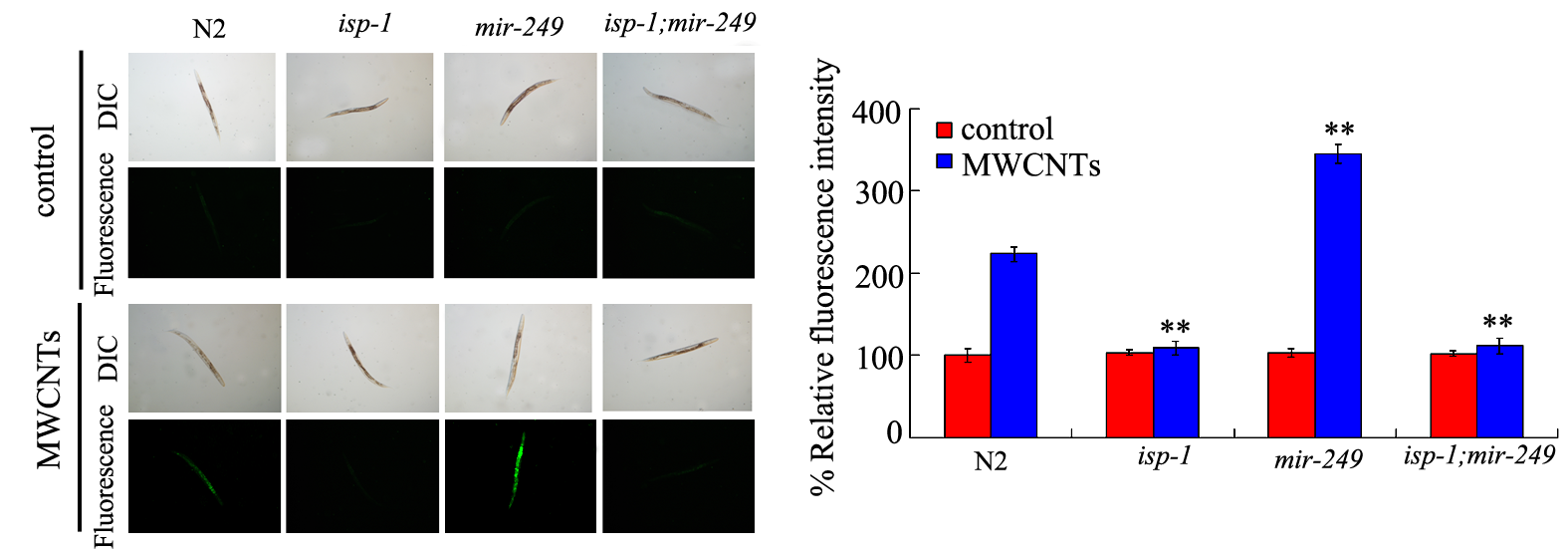


**Figure S3 | Genetic interaction of *mir-249* with ISP-1 in regulating MWCNTs toxicity on the induction of intestinal ROS production in nematodes.** The used nematode strains were wild-type N2, *mir-249(n4983)*, *isp-1(qm150)*, and *isp-1(qm150);mir-249(n4983)*. MWCNTs (1 mg/L) exposure was performed from L1-larvae to young adult. Bars represent means ± S.E.M. ^**^*P* < 0.01 *vs* N2.


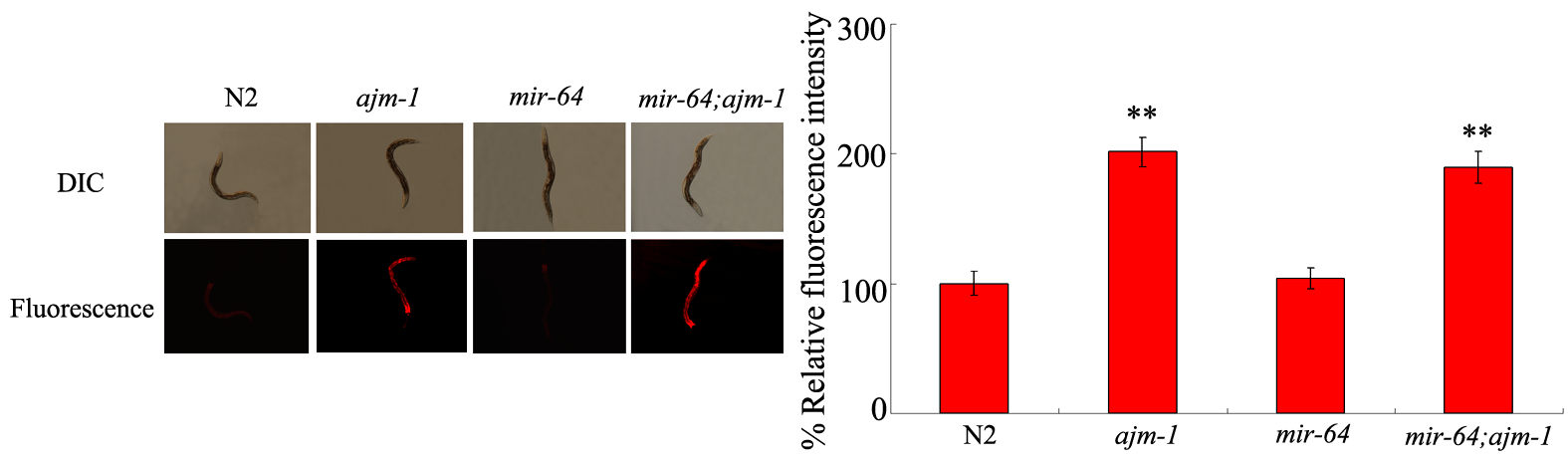


**Figure S4 | Genetic interaction of *mir-64* with AJM-1 in regulating intestinal permeability in nematodes.**  The intestinal permeability was assessed by the relative fluorescence intensity of Nile Red signals in intestine of nematodes. The used nematode strains were wild-type N2, *mir-64(nDf52)*, ajm-1(RNAi), and *mir-64(nDf52);ajm-1(RNAi)*. Bars represent means ± S.E.M. ^**^*P* < 0.01 *vs* N2.


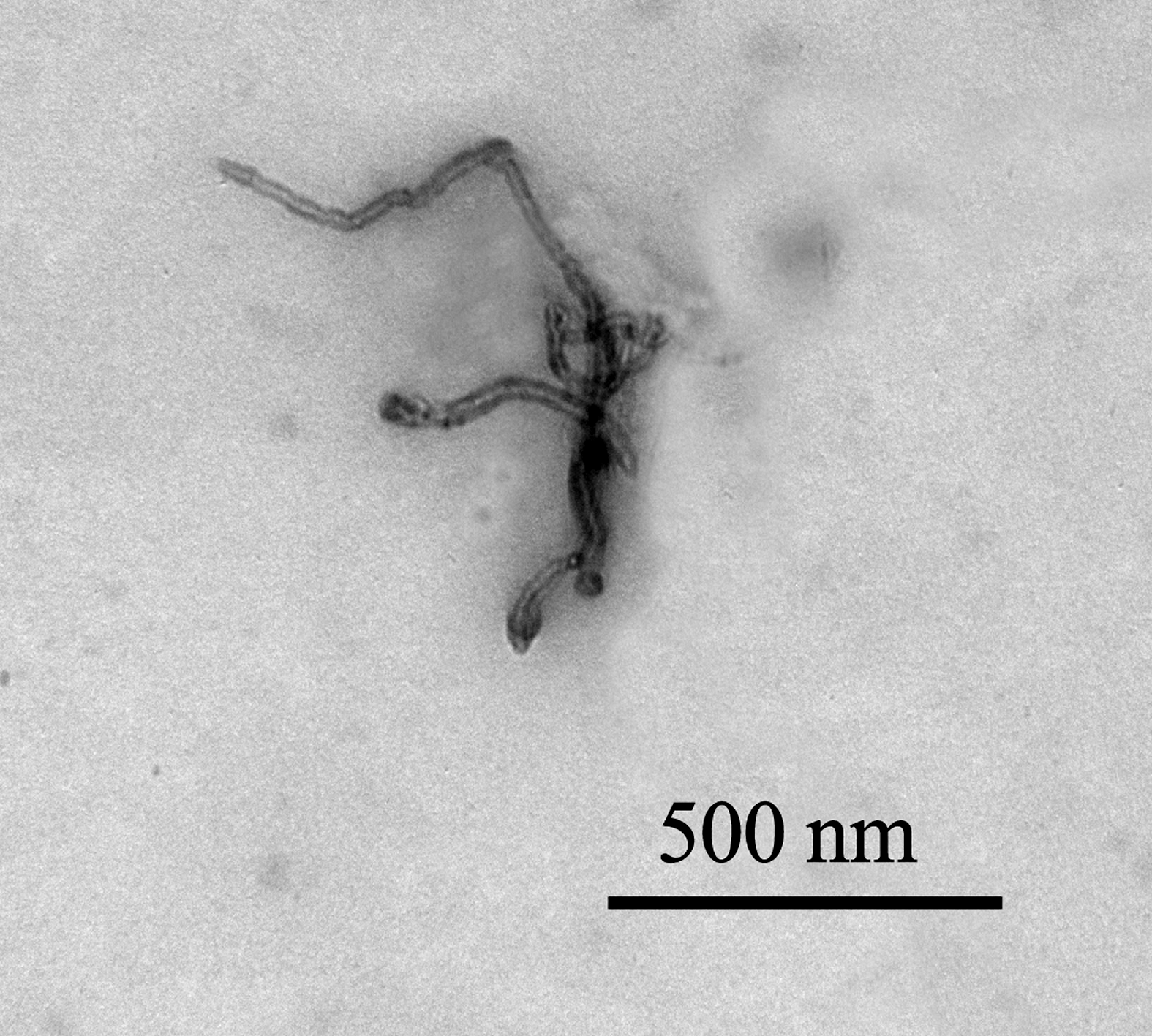


**Figure S5 | TEM picture of MWCNTs in K-medium after sonication.**

**Table S1 | The dysregulated mRNAs induced by MWCNTs exposure.**

| ID | Gene | FC | Regulation | ID | Gene | FC | Regulation |
| --- | --- | --- | --- | --- | --- | --- | --- |
| *T12C9.1* | *nhr-273* | -2.050 | down | *F41E6.8* |  | 9.885 | up |
| *K09C6.1* |  | -2.050 | down | *C27A7.4* | *che-11* | 9.220 | up |
| *ZK75.2* | *ins-2* | -2.050 | down | *R13A5.4* | *lgc-12* | 8.677 | up |
| *F29B9.12* |  | -2.051 | down | *F09C6.1* |  | 7.836 | up |
| *F28F8.2* | *acs-2* | -2.051 | down | *Y65B4BR.9* | *ceh-9* | 7.817 | up |
| *F46B3.3* | *ttr-11* | -2.051 | down | *K07E8.5* | *frpr-14* | 7.468 | up |
| *T10D4.13* | *ins-19* | -2.051 | down | *Y59E9AL.8* |  | 7.343 | up |
| *C08E3.5* | *fbxa-162* | -2.052 | down | *F14D7.10* |  | 7.342 | up |
| *F31D5.7* |  | -2.052 | down | *Y39A1A.18* |  | 6.749 | up |
| *C07G3.2* | *irg-1* | -2.052 | down | *F08D12.8* | *fbxb-105* | 6.679 | up |
| *F48C1.11* |  | -2.054 | down | *ZK1290.2* | *tph-1* | 6.570 | up |
| *T05E8.1* | *ferl-1* | -2.056 | down | *T20H9.6* |  | 6.506 | up |
| *Y6E2A.5* |  | -2.058 | down | *F31B12.4* |  | 6.471 | up |
| *W08F4.11* |  | -2.058 | down | *C35D10.12* |  | 6.218 | up |
| *Y110A7A.2* |  | -2.059 | down | *T13C5.2* |  | 6.126 | up |
| *F13E9.15* |  | -2.059 | down | *T21C9.7* | *srg-32* | 5.876 | up |
| *C09H10.4* |  | -2.059 | down | *C10F3.7* |  | 5.827 | up |
| *F11C1.9* |  | -2.059 | down | *C54F6.8* | *nhr-171* | 5.827 | up |
| *Y17D7B.3* |  | -2.060 | down | *T27A1.3* |  | 5.827 | up |
| *Y67D8C.6* |  | -2.061 | down | *F36H5.13* |  | 5.827 | up |
| *H10D12.2* |  | -2.061 | down | *Y71G12B.33* |  | 5.827 | up |
| *T04B2.8* |  | -2.062 | down | *F35C5.7* | *clec-64* | 5.827 | up |
| *T19C3.7* |  | -2.062 | down | *F07C6.6* |  | 5.827 | up |
| *Y39B6A.8* |  | -2.062 | down | *ZK643.6* |  | 5.800 | up |
| *F37C12.21* |  | -2.062 | down | *Y49E10.9* | *wht-9* | 5.482 | up |
| *F46F2.4* |  | -2.062 | down | *T23F2.4* |  | 5.440 | up |
| *M02F4.1* |  | -2.062 | down | *Y41D4B.6* |  | 5.393 | up |
| *C06A5.2* |  | -2.062 | down | *B0222.11* |  | 5.329 | up |
| *F23D12.5* | *jmjd-3.2* | -2.063 | down | *Y63D3A.10* | *fbxb-56* | 5.202 | up |
| *Y110A7A.20* | *ift-20* | -2.063 | down | *C56E6.4* |  | 5.149 | up |
| *ZK455.7* | *pgp-3* | -2.064 | down | *Y47D7A.11* |  | 5.149 | up |
| *Y55F3BR.11* |  | -2.065 | down | *F07B10.4* |  | 5.149 | up |
| *F43C1.7* |  | -2.066 | down | *C17C3.5* |  | 5.149 | up |
| *F35B12.4* |  | -2.066 | down | *F58E1.8* | *fbxb-18* | 5.149 | up |
| *F22E5.8* |  | -2.067 | down | *F14H12.2* |  | 5.149 | up |
| *F47H4.10* | *skr-5* | -2.067 | down | *T21B4.9* | *srh-70* | 5.141 | up |
| *W01C9.2* |  | -2.068 | down | *R11G1.1* |  | 5.089 | up |
| *C33F10.13* | *fbxb-88* | -2.068 | down | *B0478.3* |  | 5.056 | up |
| *C33E10.8* |  | -2.068 | down | *ZK262.2* |  | 5.041 | up |
| *F58H7.5* |  | -2.068 | down | *F45B8.4* | *pag-3* | 4.863 | up |
| *D1079.1* |  | -2.068 | down | *Y38E10A.18* | *nhr-234* | 4.849 | up |
| *F12E12.10* | *fbxb-90* | -2.068 | down | *F36D3.9* | *cpr-2* | 4.837 | up |
| *C18D11.9* |  | -2.068 | down | *C17H1.8* |  | 4.810 | up |
| *K07E12.2* |  | -2.068 | down | *Y39F10C.2* |  | 4.739 | up |
| *M04G12.3* | *gcy-34* | -2.068 | down | *Y75B8A.21* | *fbxa-89* | 4.720 | up |
| *Y38F2AL.6* |  | -2.068 | down | *T05A1.5* |  | 4.678 | up |
| *C09E7.5* |  | -2.070 | down | *F09G8.10* |  | 4.657 | up |
| *C35C5.5* | *lev-8* | -2.070 | down | *Y19D10B.4* |  | 4.645 | up |
| *F47B7.6* |  | -2.070 | down | *Y40B10B.1* |  | 4.627 | up |
| *Y41D4B.10* | *dsl-3* | -2.071 | down | *C34E11.7* |  | 4.613 | up |
| *F33D11.5* | *twk-47* | -2.072 | down | *F56C3.4* |  | 4.588 | up |
| *F45G2.5* | *bli-5* | -2.072 | down | *F29D10.2* |  | 4.575 | up |
| *Y41D4B.8* | *nhr-92* | -2.072 | down | *R04B5.1* |  | 4.514 | up |
| *C36B7.8* |  | -2.073 | down | *Y57E12B.4* |  | 4.470 | up |
| *Y23H5B.7* |  | -2.073 | down | *F14F7.5* |  | 4.470 | up |
| *C44C1.6* |  | -2.075 | down | *F44B9.9* |  | 4.470 | up |
| *C50E3.16* | *folt-3* | -2.075 | down | *T19H12.4* | *srd-33* | 4.470 | up |
| *ZK84.6* | *ins-6* | -2.075 | down | *C02H7.2* | *npr-19* | 4.470 | up |
| *Y73C8C.4* |  | -2.077 | down | *Y105C5B.8* |  | 4.470 | up |
| *F49E12.8* |  | -2.077 | down | *R11G11.7* | *pqn-60* | 4.470 | up |
| *T13C2.7* |  | -2.077 | down | *F08D12.9* | *sdz-10* | 4.470 | up |
| *F28C6.10* |  | -2.079 | down | *B0207.5* |  | 4.469 | up |
| *ZC15.10* |  | -2.080 | down | *R52.6* |  | 4.457 | up |
| *E02C12.4* | *ttr-40* | -2.081 | down | *Y47D3B.3* |  | 4.423 | up |
| *Y22D7AL.6* |  | -2.082 | down | *F10A3.3* | *fbxa-18* | 4.414 | up |
| *C03E10.6* | *clec-222* | -2.083 | down | *F36D1.2* | *sre-22* | 4.410 | up |
| *Y54E2A.5* |  | -2.084 | down | *K02E11.11* |  | 4.404 | up |
| *C52D10.9* | *skr-8* | -2.084 | down | *C11E4.3* | *tag-263* | 4.387 | up |
| *C08B6.15* |  | -2.085 | down | *T08G3.10* | *srw-35* | 4.272 | up |
| *R09B5.13* | *cnc-11* | -2.085 | down | *W03B1.5* |  | 4.269 | up |
| *Y6G8.9* |  | -2.085 | down | *T28F4.2* | *asic-2* | 4.225 | up |
| *B0238.15* |  | -2.086 | down | *Y53H1A.7* |  | 4.191 | up |
| *C37A5.4* | *fipr-23* | -2.087 | down | *Y43D4A.8* |  | 4.160 | up |
| *C17F4.12* |  | -2.088 | down | *C16B8.8* |  | 4.146 | up |
| *ZK512.1* |  | -2.088 | down | *Y37E11AR.7* |  | 4.131 | up |
| *T28C12.6* |  | -2.088 | down | *W06D11.4* |  | 4.131 | up |
| *F12E12.4* | *bath-31* | -2.089 | down | *Y37B11A.4* |  | 4.102 | up |
| *Y41D4B.26* |  | -2.089 | down | *K08F4.12* |  | 4.067 | up |
| *F35F10.13* |  | -2.089 | down | *F54E7.5* | *sdz-21* | 4.061 | up |
| *T10B9.9* |  | -2.091 | down | *ZK488.6* |  | 4.058 | up |
| *F16D3.7* | *ser-5* | -2.091 | down | *K08E3.2* |  | 4.025 | up |
| *ZK287.8* | *her-1* | -2.093 | down | *K10C9.9* |  | 4.018 | up |
| *B0244.4* |  | -2.093 | down | *F46F5.16* |  | 3.995 | up |
| *C54G6.2* |  | -2.094 | down | *F53G2.2* |  | 3.989 | up |
| *ZC374.1* | *gnrr-3* | -2.095 | down | *Y38E10A.16* | *nspe-5* | 3.978 | up |
| *H24O09.2* | *fbxb-72* | -2.095 | down | *ZK616.1* |  | 3.962 | up |
| *H02I12.4* | *dsl-6* | -2.096 | down | *T20D4.16* |  | 3.898 | up |
| *Y73F8A.15* |  | -2.096 | down | *E04A4.2* | *fbxb-80* | 3.880 | up |
| *T13C5.7* |  | -2.098 | down | *ZK1248.17* |  | 3.880 | up |
| *C31A11.7* | *oac-7* | -2.098 | down | *W05B5.2* | *npr-14* | 3.869 | up |
| *AH10.4* |  | -2.098 | down | *C43H6.9* | *glr-7* | 3.864 | up |
| *F56F10.2* |  | -2.099 | down | *Y54G2A.48* |  | 3.829 | up |
| *F10G2.2* |  | -2.101 | down | *C02E7.1* | *catp-2* | 3.811 | up |
| *R03G5.2* | *sek-1* | -2.101 | down | *F46B3.22* |  | 3.810 | up |
| *K07F5.16* |  | -2.104 | down | *C43D7.5* | *sdz-6* | 3.792 | up |
| *T04F8.9* |  | -2.105 | down | *ZK563.2* |  | 3.792 | up |
| *B0478.1* | *jnk-1* | -2.105 | down | *R09D1.1* |  | 3.792 | up |
| *C24H11.4* | *srd-74* | -2.105 | down | *K09D9.9* |  | 3.792 | up |
| *E03A3.5* |  | -2.105 | down | *ZK1055.5* |  | 3.792 | up |
| *K02E7.5* |  | -2.105 | down | *F21F12.1* |  | 3.792 | up |
| *K12B6.11* |  | -2.106 | down | *F44A6.3* |  | 3.792 | up |
| *Y47H9C.2* | *dhhc-2* | -2.107 | down | *Y67D8C.7* |  | 3.792 | up |
| *Y75B8A.33* |  | -2.107 | down | *Y60A9.3* |  | 3.792 | up |
| *B0218.3* | *pmk-1* | -2.107 | down | *ZK550.1* | *mboa-5* | 3.792 | up |
| *Y82E9BL.12* |  | -2.108 | down | *C25F9.2* |  | 3.792 | up |
| *ZK6.3* | *scl-27* | -2.108 | down | *C08H9.12* |  | 3.792 | up |
| *ZK430.5* |  | -2.110 | down | *C07E3.8* |  | 3.792 | up |
| *C33G8.10* | *nhr-162* | -2.110 | down | *F08D12.11* | *fbxb-111* | 3.792 | up |
| *Y5H2B.5* | *cyp-32B1* | -2.111 | down | *C37C3.3* | *vps-32.2* | 3.792 | up |
| *F40F12.9* |  | -2.111 | down | *T11G6.7* |  | 3.792 | up |
| *C30H6.2* | *tag-141* | -2.111 | down | *Y60A9A.1* |  | 3.792 | up |
| *Y73B6BL.35* |  | -2.111 | down | *ZK328.8* | *tbx-7* | 3.779 | up |
| *C06C3.11* |  | -2.113 | down | *C54E10.11* |  | 3.778 | up |
| *D2024.4* |  | -2.113 | down | *ZK682.7* |  | 3.775 | up |
| *C18C4.1* | *glb-5* | -2.114 | down | *F19B2.7* |  | 3.771 | up |
| *F54B8.4* |  | -2.114 | down | *F46C8.7* | *glb-16* | 3.760 | up |
| *T05C12.11* |  | -2.117 | down | *T27F6.1* |  | 3.759 | up |
| *K06B4.11* | *nhr-53* | -2.117 | down | *F16H6.4* |  | 3.733 | up |
| *C17F4.3* |  | -2.118 | down | *W01A8.3* | *cutl-6* | 3.724 | up |
| *F15A4.8* |  | -2.119 | down | *K08H10.6* |  | 3.708 | up |
| *C06C3.9* |  | -2.119 | down | *K09C6.2* |  | 3.707 | up |
| *R09H10.6* |  | -2.120 | down | *R08C7.6* | *clec-175* | 3.696 | up |
| *F56B3.9* |  | -2.120 | down | *C31G12.4* |  | 3.692 | up |
| *F20E11.17* |  | -2.122 | down | *Y75B12B.11* |  | 3.676 | up |
| *F31E9.3* |  | -2.122 | down | *F08G2.2* | *his-43* | 3.672 | up |
| *C11G10.1* |  | -2.122 | down | *F18A12.3* |  | 3.655 | up |
| *C02F5.2* |  | -2.124 | down | *F35E2.6* | *oac-19* | 3.649 | up |
| *R13H8.1* | *daf-16* | -2.124 | down | *K01A2.6* |  | 3.632 | up |
| *F15A4.2* |  | -2.124 | down | *F55B11.6* |  | 3.603 | up |
| *Y49E10.8* |  | -2.125 | down | *W01B6.11* |  | 3.592 | up |
| *B0334.13* |  | -2.126 | down | *F39D8.4* | *nas-13* | 3.559 | up |
| *Y39G10AR.6* | *ugt-31* | -2.126 | down | *ZK666.14* |  | 3.554 | up |
| *ZK550.6* |  | -2.128 | down | *E03A3.1* |  | 3.538 | up |
| *F53F4.15* |  | -2.128 | down | *H04D03.4* |  | 3.520 | up |
| *F44G4.6* |  | -2.131 | down | *ZC247.1* |  | 3.488 | up |
| *F58H7.1* |  | -2.131 | down | *Y47D7A.7* |  | 3.453 | up |
| *C15C8.5* |  | -2.132 | down | *H23N18.2* | *ugt-14* | 3.453 | up |
| *T28A11.15* | *srt-63* | -2.132 | down | *F41D3.4* | *oac-27* | 3.449 | up |
| *F41D3.6* |  | -2.133 | down | *C08E8.10* |  | 3.413 | up |
| *F17C11.13* |  | -2.133 | down | *F32A7.8* |  | 3.407 | up |
| *F56D6.10* | *dct-8* | -2.134 | down | *C31H5.3* | *acr-19* | 3.398 | up |
| *F38A5.12* | *nspb-2* | -2.135 | down | *T16A1.3* | *fbxc-49* | 3.397 | up |
| *T28F2.8* | *col-51* | -2.135 | down | *F55F1.3* |  | 3.392 | up |
| *F31D5.6* |  | -2.136 | down | *AC3.12* |  | 3.385 | up |
| *T25F10.5* | *bbs-8* | -2.136 | down | *ZK131.6* | *his-12* | 3.379 | up |
| *K02E11.6* |  | -2.136 | down | *Y71F9B.15* |  | 3.358 | up |
| *Y38E10A.9* |  | -2.137 | down | *F44A2.2* | *kvs-3* | 3.350 | up |
| *C35A5.9* | *hdac-11* | -2.139 | down | *F20B6.6* |  | 3.344 | up |
| *Y73F8A.8* | *pqn-90* | -2.139 | down | *Y17G9A.2* |  | 3.340 | up |
| *W02D7.3* |  | -2.139 | down | *ZK1290.9* |  | 3.340 | up |
| *H10D18.1* |  | -2.139 | down | *C54F6.3* |  | 3.331 | up |
| *R09F10.2* | *abu-9* | -2.140 | down | *Y19D10A.15* | *srd-75* | 3.328 | up |
| *R52.9* | *math-37* | -2.141 | down | *F56C3.8* |  | 3.295 | up |
| *F53B3.3* |  | -2.142 | down | *K07G5.4* |  | 3.293 | up |
| *W08F4.13* |  | -2.142 | down | *C55C3.8* |  | 3.284 | up |
| *ZC334.2* | *ins-30* | -2.142 | down | *C05B5.12* |  | 3.281 | up |
| *T05B4.9* |  | -2.142 | down | *C02E7.10* |  | 3.278 | up |
| *F40F4.1* | *fbxb-71* | -2.142 | down | *F44F4.6* | *gly-1* | 3.262 | up |
| *W04A8.3* |  | -2.142 | down | *Y47H9C.14* |  | 3.252 | up |
| *Y23H5B.12* |  | -2.142 | down | *Y97E10AR.8* |  | 3.244 | up |
| *M151.8* | *fbxb-33* | -2.142 | down | *F29C4.5* | *duo-2* | 3.244 | up |
| *T26C12.3* |  | -2.142 | down | *ZK973.6* | *anc-1* | 3.241 | up |
| *F43A11.8* |  | -2.142 | down | *T02B11.6* |  | 3.232 | up |
| *C53D6.12* |  | -2.142 | down | *F29C4.4* |  | 3.218 | up |
| *T04C12.3* |  | -2.142 | down | *Y57G11B.7* |  | 3.217 | up |
| *W10G11.1* |  | -2.144 | down | *T26E4.9* |  | 3.210 | up |
| *C01G12.11* | *nspb-9* | -2.145 | down | *C17H1.4* |  | 3.201 | up |
| *T12A7.1* | *gem-4* | -2.146 | down | *ZK697.6* | *gst-21* | 3.195 | up |
| *F42G9.8* | *tpst-2* | -2.146 | down | *F32A6.2* | *ift-81* | 3.195 | up |
| *ZK795.6* |  | -2.147 | down | *F36H9.8* |  | 3.189 | up |
| *F09F7.1* |  | -2.148 | down | *F30A10.11* |  | 3.183 | up |
| *W05G11.3* | *col-88* | -2.151 | down | *F02D10.3* |  | 3.180 | up |
| *C05A9.1* | *pgp-5* | -2.151 | down | *F54G8.2* | *dgk-3* | 3.179 | up |
| *T09B4.6* |  | -2.151 | down | *Y59E9AL.5* |  | 3.176 | up |
| *C49D10.11* | *sdz-7* | -2.151 | down | *ZK250.7* | *math-49* | 3.173 | up |
| *R03E9.3* | *abts-4* | -2.151 | down | *Y8A9A.2* |  | 3.169 | up |
| *W04C9.5* |  | -2.152 | down | *C02B8.3* |  | 3.160 | up |
| *T19A6.4* |  | -2.152 | down | *Y41D4B.1* |  | 3.122 | up |
| *K03H9.3* |  | -2.152 | down | *R03H4.1* | *oac-40* | 3.117 | up |
| *F37B1.1* | *gst-24* | -2.153 | down | *Y105E8A.34* |  | 3.117 | up |
| *ZK863.2* | *col-37* | -2.154 | down | *Y51A2B.6* |  | 3.116 | up |
| *F14D2.6* |  | -2.154 | down | *Y22D7AR.14* |  | 3.113 | up |
| *F08G12.11* |  | -2.155 | down | *C29F3.4* | *clec-231* | 3.113 | up |
| *F49D11.2* |  | -2.155 | down | *R13F6.8* | *clec-158* | 3.113 | up |
| *B0035.7* | *his-47* | -2.156 | down | *T10H4.4* |  | 3.113 | up |
| *Y105C5A.23* | *gnrr-8* | -2.156 | down | *Y53C12C.1* | *eyg-1* | 3.113 | up |
| *M02E1.2* |  | -2.158 | down | *C45E5.2* |  | 3.113 | up |
| *T23F4.3* |  | -2.158 | down | *F45D11.13* | *fbxb-30* | 3.113 | up |
| *F41D9.5* | *sulp-3* | -2.159 | down | *K04F1.10* |  | 3.113 | up |
| *W04G3.17* |  | -2.159 | down | *Y51A2B.2* |  | 3.113 | up |
| *C35D6.5* |  | -2.161 | down | *Y71A12C.3* |  | 3.113 | up |
| *Y44A6C.2* |  | -2.161 | down | *C12D5.10* |  | 3.113 | up |
| *C54F6.14* | *ftn-1* | -2.161 | down | *F22B8.2* |  | 3.113 | up |
| *K11G9.1* |  | -2.162 | down | *M162.8* | *fbxa-118* | 3.113 | up |
| *T09F5.9* | *clec-47* | -2.162 | down | *F22G12.3* |  | 3.113 | up |
| *Y11D7A.19* |  | -2.165 | down | *T17A3.3* | *fbxb-81* | 3.113 | up |
| *R08C7.1* |  | -2.166 | down | *Y75B8A.39* |  | 3.113 | up |
| *Y119D3A.3* | *fbxa-35* | -2.166 | down | *Y67D8B.3* |  | 3.113 | up |
| *H06A10.2* | *col-185* | -2.166 | down | *F07G11.1* |  | 3.113 | up |
| *ZC443.5* | *ugt-18* | -2.167 | down | *W01A8.7* |  | 3.113 | up |
| *T06D10.3* |  | -2.169 | down | *Y59H11AL.1* |  | 3.113 | up |
| *Y54F10BL.1* | *fbxa-42* | -2.170 | down | *F28A10.4* |  | 3.113 | up |
| *Y17D7B.2* |  | -2.173 | down | *W06H8.8* | *ttn-1* | 3.112 | up |
| *F31F7.2* |  | -2.175 | down | *Y53H1B.1* | *cutl-10* | 3.096 | up |
| *BE10.6* |  | -2.175 | down | *C42D8.9* |  | 3.092 | up |
| *T16H5.1* | *inx-19* | -2.176 | down | *F28G4.2* |  | 3.066 | up |
| *ZC239.16* |  | -2.176 | down | *K07E12.1* | *dig-1* | 3.041 | up |
| *R06B9.1* | *arrd-11* | -2.176 | down | *C32C4.2* | *aqp-6* | 3.015 | up |
| *F54D10.8* |  | -2.177 | down | *ZK856.14* |  | 3.013 | up |
| *Y43F8A.1* |  | -2.180 | down | *C23H5.3* | *xbx-4* | 3.010 | up |
| *D2096.9* |  | -2.181 | down | *R166.7* |  | 3.010 | up |
| *Y51H4A.9* | *col-137* | -2.181 | down | *F09F3.11* | *srx-135* | 3.010 | up |
| *Y41D4B.24* |  | -2.181 | down | *F31A9.4* | *fbxc-37* | 3.010 | up |
| *Y49E10.7* |  | -2.182 | down | *Y51B9A.7* |  | 3.007 | up |
| *C32H11.11* |  | -2.183 | down | *M01H9.2* |  | 3.006 | up |
| *W08F4.5* |  | -2.183 | down | *F35H12.7* |  | 3.004 | up |
| *Y60C6A.1* |  | -2.184 | down | *ZK1025.7* | *bah-1* | 2.998 | up |
| *F53G12.7* | *col-45* | -2.184 | down | *Y14H12A.2* |  | 2.996 | up |
| *C49A9.8* | *ugt-24* | -2.186 | down | *F35E2.10* |  | 2.987 | up |
| *Y40B10A.6* |  | -2.186 | down | *F53F4.6* | *rdy-2* | 2.986 | up |
| *Y19D10A.11* |  | -2.187 | down | *T23F1.5* |  | 2.986 | up |
| *C53D6.18* |  | -2.188 | down | *T13A10.1* |  | 2.982 | up |
| *T20D4.3* |  | -2.188 | down | *T22B7.5* | *srv-7* | 2.979 | up |
| *Y6G8.5* |  | -2.189 | down | *F42E8.2* |  | 2.978 | up |
| T07A9.6 | *daf-18* | -2.189 | down | *Y39H10A.2* | *oac-55* | 2.976 | up |
| *F53C3.3* |  | -2.190 | down | *T08G5.15* |  | 2.969 | up |
| *Y57G11B.97* |  | -2.190 | down | *ZK816.5* | *dhs-26* | 2.966 | up |
| *Y53G8AL.4* |  | -2.192 | down | *Y75B8A.10* |  | 2.965 | up |
| *F35G2.12* |  | -2.192 | down | *C44B12.6* |  | 2.963 | up |
| *Y58A7A.5* |  | -2.193 | down | *D1007.13* |  | 2.962 | up |
| *F53F8.1* | *klf-2* | -2.193 | down | *ZK816.1* |  | 2.960 | up |
| *Y113G7B.27* |  | -2.194 | down | *C06E4.6* |  | 2.949 | up |
| *C25D7.4* | *fbxa-82* | -2.195 | down | *Y54F10BM.6* |  | 2.944 | up |
| *F26D10.11* |  | -2.195 | down | *K06C4.9* | *frpr-12* | 2.944 | up |
| *W09G10.3* |  | -2.197 | down | *F58E1.10* | *fbxc-22* | 2.944 | up |
| *T13B5.5* | *lips-11* | -2.197 | down | *C04F5.7* | *ugt-63* | 2.935 | up |
| *F38A5.14* | *nspb-1* | -2.198 | down | *ZK262.7* | *srw-82* | 2.921 | up |
| *F38E11.1* | *hsp-12.3* | -2.199 | down | *Y41C4A.17* |  | 2.910 | up |
| *R10D12.18* |  | -2.200 | down | *T23G5.5* | *dat-1* | 2.898 | up |
| *W05B2.4* |  | -2.201 | down | *M03E7.4* |  | 2.885 | up |
| *C30A5.4* |  | -2.201 | down | *R06B10.4* | *trp-2* | 2.880 | up |
| *T16G1.5* |  | -2.202 | down | *K01A11.3* |  | 2.875 | up |
| *K07H8.7* |  | -2.203 | down | *ZC168.2* |  | 2.874 | up |
| *ZK666.15* |  | -2.203 | down | *C03A3.3* |  | 2.866 | up |
| *C27C7.3* | *nhr-74* | -2.203 | down | *R05G9R.1* |  | 2.860 | up |
| *K11G9.6* | *mtl-1* | -2.205 | down | *C34B4.7* |  | 2.852 | up |
| *C45B2.8* |  | -2.206 | down | *W03D8.5* |  | 2.851 | up |
| *C30G12.1* |  | -2.206 | down | *C33C12.7* |  | 2.846 | up |
| *C06C3.8* |  | -2.207 | down | *T19H12.10* | *ugt-11* | 2.841 | up |
| *D2096.13* |  | -2.207 | down | *Y102A5C.16* | *clec-239* | 2.834 | up |
| *C43F9.11* |  | -2.208 | down | *C53D6.9* | *srx-15* | 2.833 | up |
| *K08A8.1* | *mek-1* | -2.209 | down | *Y105C5B.29* | *hlh-32* | 2.831 | up |
| *F07B7.8* |  | -2.209 | down | *ZK384.5* |  | 2.824 | up |
| *T07D1.1* | *gcy-31* | -2.210 | down | *F10G2.7* |  | 2.823 | up |
| *F20C5.7* |  | -2.210 | down | *T25B6.3* |  | 2.821 | up |
| *B0250.2* | *calf-1* | -2.211 | down | *Y50E8A.6* |  | 2.817 | up |
| *C25A11.4* | *ajm-1* | -2.212 | down | *F59D12.2* |  | 2.811 | up |
| *F38H4.15* |  | -2.212 | down | *Y82E9BL.19* |  | 2.807 | up |
| *K02E11.3* |  | -2.213 | down | *M195.2* |  | 2.806 | up |
| *C11E4.8* |  | -2.214 | down | *F19F10.1* |  | 2.805 | up |
| *R02D5.4* |  | -2.214 | down | *C02B8.4* | *hlh-8* | 2.799 | up |
| *C30H6.12* |  | -2.216 | down | *F19B10.8* | *srx-98* | 2.798 | up |
| *ZC190.8* |  | -2.216 | down | *F38A1.4* | *clec-167* | 2.788 | up |
| *C32E8.1* |  | -2.216 | down | *F55C10.4* |  | 2.786 | up |
| *K10G4.10* |  | -2.216 | down | *Y45F10B.10* | *qui-1* | 2.785 | up |
| *F53C3.8* |  | -2.220 | down | *F54E2.4* |  | 2.784 | up |
| *F59D12.1* |  | -2.221 | down | *F41E6.17* |  | 2.782 | up |
| *F20D12.3* | *bbs-2* | -2.222 | down | *D2024.10* |  | 2.781 | up |
| *F46A9.2* |  | -2.222 | down | *R11G11.6* |  | 2.778 | up |
| *F19B10.13* |  | -2.222 | down | *C09E7.10* |  | 2.774 | up |
| *T01H10.2* | *lgc-14* | -2.222 | down | *F25H9.3* |  | 2.774 | up |
| *K03D7.7* | *fbxa-102* | -2.223 | down | *F56A11.4* |  | 2.774 | up |
| *C14A6.15* |  | -2.223 | down | *C08H9.14* |  | 2.774 | up |
| *K02F6.8* |  | -2.223 | down | *F18E2.4* | *srt-43* | 2.774 | up |
| *C36C9.4* |  | -2.223 | down | *K08D12.4* |  | 2.774 | up |
| *T27A8.3* |  | -2.223 | down | *F35H10.3* |  | 2.770 | up |
| *F15E6.8* | *dct-7* | -2.226 | down | *K10G6.1* | *lin-31* | 2.767 | up |
| *F56A4.4* |  | -2.226 | down | *F28H7.7* |  | 2.766 | up |
| *Y57G11C.1140* |  | -2.227 | down | *C32B5.5* | *fbxc-14* | 2.766 | up |
| *Y37E3.24* |  | -2.228 | down | *Y52B11A.18* |  | 2.765 | up |
| *R09B5.2* | *cnc-1* | -2.229 | down | *C05C8.8* |  | 2.765 | up |
| *F11A6.2* | *scrm-4* | -2.233 | down | *Y51A2D.5* | *hmit-1.2* | 2.764 | up |
| *ZC47.10* | *fbxa-34* | -2.234 | down | *ZK265.8* | *cutl-8* | 2.759 | up |
| *C49G7.1* |  | -2.235 | down | *AH6.2* | *sfxn-1.1* | 2.755 | up |
| *K02E7.6* |  | -2.235 | down | *T22E5.3* |  | 2.754 | up |
| *F38E1.5* | *gpa-2* | -2.235 | down | *C50H2.2* | *egl-47* | 2.753 | up |
| *F59A7.2* |  | -2.236 | down | *C14C6.3* |  | 2.746 | up |
| *ZC412.13* |  | -2.236 | down | *T22B11.3* |  | 2.745 | up |
| *T20D4.17* |  | -2.237 | down | *F15B9.11* |  | 2.743 | up |
| *ZC455.3* | *ugt-3* | -2.237 | down | *F15H9.7* |  | 2.741 | up |
| *T03F7.5* |  | -2.237 | down | *M04C9.2* |  | 2.740 | up |
| *T22H6.7* | *abf-6* | -2.239 | down | *B0280.15* | *sls-2.16* | 2.739 | up |
| *cTel3X.1* |  | -2.239 | down | *Y119C1B.3* |  | 2.737 | up |
| *E04D5.2* |  | -2.240 | down | *C11E4.2* |  | 2.734 | up |
| *W08A12.2* |  | -2.240 | down | *K02F6.1* |  | 2.729 | up |
| *H10E21.2* |  | -2.240 | down | *R144.1* | *klp-6* | 2.729 | up |
| *Y82E9BL.17* | *fbxa-27* | -2.242 | down | *T19H12.6* |  | 2.726 | up |
| *F53F8.7* |  | -2.243 | down | *F59B8.1* |  | 2.725 | up |
| *C36B1.13* |  | -2.244 | down | *Y58G8A.5* |  | 2.722 | up |
| *ZK993.1* | *ceh-45* | -2.245 | down | *T20B3.11* | *clec-30* | 2.718 | up |
| *E03H12.3* | *clec-176* | -2.246 | down | *F31C3.10* | *rrn-3.56* | 2.710 | up |
| *T23F4.4* | *nas-27* | -2.247 | down | *F31C3.9* | *rrn-3.1* | 2.705 | up |
| *C26C6.7* | *glb-8* | -2.248 | down | *K09C8.6* | *nlp-19* | 2.697 | up |
| *C32E12.2* | *pde-5* | -2.249 | down | *F21F8.6* |  | 2.694 | up |
| *K09A11.5* | *phf-33* | -2.250 | down | *ZK1236.8* |  | 2.691 | up |
| *Y18H1A.8* |  | -2.251 | down | *C27F2.2* | *nca-2* | 2.686 | up |
| *ZC47.9* | *fbxa-33* | -2.253 | down | *T14E8.4* |  | 2.684 | up |
| *F18E2.5* | *gpa-13* | -2.255 | down | *R10E11.7* | *srxa-10* | 2.679 | up |
| *Y71F9AL.6* |  | -2.255 | down | *H34I24.1* |  | 2.677 | up |
| *ZK380.t2* |  | -2.255 | down | *ZK1086.2* |  | 2.676 | up |
| *C32H11.1* |  | -2.256 | down | *Y68A4A.12* |  | 2.676 | up |
| *Y81G3A.6* |  | -2.256 | down | *C32C4.1* |  | 2.672 | up |
| *F09G2.6* | *ugt-36* | -2.256 | down | *Y102A5B.1* | *clec-27* | 2.672 | up |
| *D1014.6* |  | -2.257 | down | *F34H10.4* |  | 2.670 | up |
| *F59A7.10* |  | -2.257 | down | *F56D5.3* |  | 2.666 | up |
| *Y73C8B.4* | *lag-2* | -2.257 | down | *C54F6.6* |  | 2.663 | up |
| *Y17G7B.23* |  | -2.258 | down | *Y73C8C.8* |  | 2.661 | up |
| *Y40H7A.11* |  | -2.259 | down | *K08C9.5* |  | 2.661 | up |
| *C18A11.5* | *xol-1* | -2.259 | down | *ZC47.11* |  | 2.656 | up |
| *F31D5.1* |  | -2.259 | down | *F38G1.2* | *egl-17* | 2.655 | up |
| *K09A11.2* | *cyp-14A1* | -2.260 | down | *F11A3.4* |  | 2.655 | up |
| *T08B6.2* |  | -2.260 | down | *K08D10.5* |  | 2.648 | up |
| *F56A8.1* | *anoh-1* | -2.261 | down | *K05F1.7* | *msp-63* | 2.646 | up |
| *C39B5.10* |  | -2.261 | down | *Y106G6G.6* |  | 2.641 | up |
| *T01C3.11* |  | -2.263 | down | *F58G6.4* | *acc-1* | 2.641 | up |
| *C34E10.9* |  | -2.264 | down | *Y2C2A.1* | *tag-80* | 2.640 | up |
| *T05A7.11* |  | -2.264 | down | *F52G3.5* |  | 2.637 | up |
| *Y55H10A.2* |  | -2.265 | down | *F09F3.8* |  | 2.628 | up |
| *C17B7.3* |  | -2.265 | down | *K01A12.3* |  | 2.627 | up |
| *F19D8.1* | *twk-23* | -2.265 | down | *Y87G2A.20* |  | 2.624 | up |
| *T23F4.1* |  | -2.265 | down | *H04J21.1* |  | 2.622 | up |
| *F57B1.1* |  | -2.265 | down | *R11G10.2* |  | 2.621 | up |
| *ZK616.9* | *spp-7* | -2.265 | down | *Y71A12B.23* |  | 2.617 | up |
| *Y23B4A.1* |  | -2.265 | down | *T27C10.4* | *bath-25* | 2.616 | up |
| *ZC334.3* | *ins-24* | -2.265 | down | *Y13C8A.2* |  | 2.614 | up |
| *T08D10.4* |  | -2.265 | down | *Y39G10AR.25* | *peel-1* | 2.613 | up |
| *F47H4.11* | *fbxa-134* | -2.265 | down | *Y102A5C.15* | *srh-206* | 2.610 | up |
| *T13H10.2* |  | -2.265 | down | *C27D6.14* |  | 2.608 | up |
| *B0284.5* |  | -2.265 | down | *R13A1.8* | *glb-23* | 2.604 | up |
| *H04M03.11* |  | -2.266 | down | *B0304.7* | *sra-34* | 2.600 | up |
| *F16F9.3* |  | -2.266 | down | *R02E12.5* |  | 2.599 | up |
| *B0348.1* |  | -2.267 | down | *C27H5.7* | *dyf-13* | 2.599 | up |
| *C38D4.7* |  | -2.271 | down | *F13E9.16* |  | 2.596 | up |
| *C12D5.2* | *nhr-152* | -2.271 | down | *R02D1.1* | *nhr-125* | 2.595 | up |
| *ZC334.13* |  | -2.272 | down | *C45B11.6* |  | 2.595 | up |
| *F08D12.12* |  | -2.273 | down | *R03D7.5* |  | 2.595 | up |
| *F07B7.9* | *his-50* | -2.274 | down | B0334.8 | *age-1* | 2.594 | up |
| *C52D10.10* |  | -2.274 | down | *F15H10.8* |  | 2.591 | up |
| *M162.2* | *clec-258* | -2.275 | down | *F45E1.2* |  | 2.590 | up |
| *F22F4.2* | *inx-3* | -2.275 | down | *Y50D7A.5* | *hpo-38* | 2.590 | up |
| *F09E5.1* | *pkc-3* | -2.276 | down | *C39E6.6* | *npr-1* | 2.588 | up |
| *C24G7.1* |  | -2.278 | down | *F55G11.9* | *abt-3* | 2.588 | up |
| *C14A6.2* |  | -2.278 | down | *F16B4.5* |  | 2.587 | up |
| *C06A12.5* | *lact-6* | -2.278 | down | *C17B7.8* |  | 2.586 | up |
| *M04D8.3* | *ins-23* | -2.281 | down | *Y53F4B.17* |  | 2.585 | up |
| *W04H10.5* |  | -2.283 | down | *T13A10.12* | *srv-32* | 2.581 | up |
| *C08E3.6* | *fbxa-163* | -2.284 | down | *B0198.3* |  | 2.576 | up |
| *R02C2.6* |  | -2.285 | down | *K08C9.8* |  | 2.573 | up |
| *C15H11.11* |  | -2.287 | down | *K06H7.1* |  | 2.572 | up |
| *F40F11.4* |  | -2.287 | down | *F52F12.5* |  | 2.571 | up |
| *F55C12.6* |  | -2.289 | down | *Y46G5A.28* |  | 2.571 | up |
| *Y47H9B.2* |  | -2.290 | down | *F35E8.11* | *cdr-1* | 2.570 | up |
| *B0047.4* | *math-1* | -2.291 | down | *B0554.4* |  | 2.569 | up |
| *B0414.2* | *rnt-1* | -2.291 | down | *F08D12.6* | *fbxb-108* | 2.562 | up |
| *F15H9.1* |  | -2.292 | down | *F32H2.12* |  | 2.554 | up |
| *T08G5.12* | *ins-10* | -2.295 | down | *C06G1.2* |  | 2.553 | up |
| *C02D5.3* | *gsto-2* | -2.296 | down | *C18C4.9* | *glb-6* | 2.552 | up |
| *Y105C5B.16* | *lgc-19* | -2.297 | down | *K07F5.6* |  | 2.546 | up |
| *Y51A2D.11* | *ttr-26* | -2.298 | down | *F28F5.4* |  | 2.546 | up |
| *F11A6.15* |  | -2.300 | down | *R13A1.5* |  | 2.544 | up |
| *C06B8.4* | *str-264* | -2.303 | down | *T28A11.17* |  | 2.541 | up |
| *C07H6.9* |  | -2.303 | down | *Y38H6C.14* |  | 2.541 | up |
| *T27E9.6* |  | -2.303 | down | *Y67D8C.4* |  | 2.540 | up |
| *Y50E8A.5* |  | -2.304 | down | *F58E1.6* | *nhx-6* | 2.540 | up |
| *F54E7.3* | *par-3* | -2.305 | down | *Y38E10A.13* | *nspe-1* | 2.539 | up |
| *K04C2.5* |  | -2.306 | down | *E02H4.2* |  | 2.538 | up |
| *M03B6.5* |  | -2.306 | down | *H06I04.9* |  | 2.533 | up |
| *Y82E9BL.13* | *fbxa-79* | -2.306 | down | *C41C4.2* | *sre-2* | 2.529 | up |
| *T28F2.6* | *col-50* | -2.306 | down | *R05D8.7* |  | 2.527 | up |
| *R09E10.4* |  | -2.306 | down | *R03H4.6* | *bus-1* | 2.526 | up |
| *Y57G11C.35* |  | -2.307 | down | *K07D4.6* |  | 2.525 | up |
| *T22H2.3* | *sri-11* | -2.309 | down | *ZC412.7* | *nspa-3* | 2.521 | up |
| *F02C12.8* |  | -2.310 | down | *Y18D10A.7* | *ptr-17* | 2.519 | up |
| *C49G7.5* | *irg-2* | -2.312 | down | *T10C6.11* | *his-4* | 2.519 | up |
| *C16C10.13* |  | -2.313 | down | *F55C9.5* |  | 2.510 | up |
| *Y48G8AR.2* |  | -2.314 | down | *F15B9.7* | *fmi-1* | 2.509 | up |
| *R10E8.4* |  | -2.316 | down | *F14H12.6* |  | 2.507 | up |
| *Y37B11A.6* |  | -2.316 | down | *K06A4.6* |  | 2.504 | up |
| *F45D11.10* | *fbxc-39* | -2.316 | down | *F28B4.1* |  | 2.502 | up |
| *C38D9.9* | *fbxa-176* | -2.318 | down | *DY3.3* | *hlh-16* | 2.498 | up |
| *C08E3.4* | *fbxa-161* | -2.318 | down | *C14A11.2* |  | 2.494 | up |
| *C25F9.16* |  | -2.319 | down | *R02C2.1* |  | 2.490 | up |
| *R09D1.6* |  | -2.322 | down | *T27E4.5* |  | 2.486 | up |
| *H24K24.2* |  | -2.323 | down | *F31C3.13* |  | 2.478 | up |
| *Y41D4B.15* |  | -2.324 | down | *C26C6.6* |  | 2.477 | up |
| *K05F6.4* |  | -2.326 | down | *T02G6.2* |  | 2.475 | up |
| *W01A8.10* |  | -2.326 | down | *C44C11.6* |  | 2.473 | up |
| *T07A5.4* | *ostf-4* | -2.329 | down | *T25C8.1* |  | 2.472 | up |
| *C33D12.7* | *ceh-30* | -2.330 | down | *Y80D3A.7* | *ptr-22* | 2.467 | up |
| *C43C3.4* |  | -2.331 | down | *C09E8.4* |  | 2.465 | up |
| *T13B5.4* | *col-40* | -2.331 | down | *F35H12.1* |  | 2.465 | up |
| *C08G5.6* |  | -2.332 | down | *R11H6.7* |  | 2.464 | up |
| *C06B8.1* | *nhr-150* | -2.339 | down | *F53F10.6* |  | 2.462 | up |
| *C07B5.2* |  | -2.340 | down | *W08A12.4* |  | 2.460 | up |
| *Y113G7B.6* | *fbxa-113* | -2.342 | down | *F49A5.3* | *clec-22* | 2.457 | up |
| *R10D12.9* | *swt-6* | -2.344 | down | *F49B2.4* |  | 2.455 | up |
| *Y59E9AR.6* | *thn-7* | -2.345 | down | *Y46H3D.1* |  | 2.455 | up |
| *F08G2.3* | *his-42* | -2.346 | down | *W06A7.5* | *nspa-8* | 2.454 | up |
| *Y41G9A.3* | *aakg-3* | -2.348 | down | *T19D12.7* |  | 2.453 | up |
| *F31F4.14* | *sru-25* | -2.349 | down | *C48E7.9* | *twk-37* | 2.451 | up |
| *ZC239.14* |  | -2.349 | down | *ZC21.3* |  | 2.451 | up |
| *F10D2.15* |  | -2.349 | down | *F26A1.8* |  | 2.450 | up |
| *R09F10.7* | *pqn-57* | -2.350 | down | *F49A5.9* | *clec-32* | 2.440 | up |
| *F10D2.13* |  | -2.353 | down | *F09E5.12* |  | 2.438 | up |
| *K08E7.4* |  | -2.353 | down | *C26C6.8* |  | 2.437 | up |
| *F55D12.4* | *unc-55* | -2.354 | down | *F46B6.2* |  | 2.435 | up |
| *F10D2.8* |  | -2.355 | down | *C12D5.11* | *sre-11* | 2.435 | up |
| *Y61B8B.1* | *sri-70* | -2.358 | down | *F17A2.3* | *phf-32* | 2.435 | up |
| *W04G5.11* |  | -2.358 | down | *R03H4.5* | *oac-41* | 2.435 | up |
| *R148.1* | *mks-1* | -2.359 | down | *W07A12.5* | *col-78* | 2.435 | up |
| *T22G5.4* | *srsx-19* | -2.361 | down | *Y75B7B.2* |  | 2.435 | up |
| *C39B10.1* |  | -2.363 | down | *F44E5.3* |  | 2.435 | up |
| *T22B2.1* |  | -2.363 | down | *T05E11.7* |  | 2.435 | up |
| *F53G2.1* |  | -2.363 | down | *F16B12.7* |  | 2.435 | up |
| *W05B10.6* |  | -2.363 | down | *Y116A8C.7* |  | 2.435 | up |
| *F15A4.13* | *fbxb-102* | -2.363 | down | *F31F4.4* | *srx-21* | 2.435 | up |
| *C29F3.6* | *srx-58* | -2.363 | down | *F52D4.1* |  | 2.435 | up |
| *F42G8.12* | *isp-1* | -2.366 | down | *F55C9.7* | *fbxb-60* | 2.435 | up |
| *T01C1.4* |  | -2.366 | down | *R09E10.9* |  | 2.435 | up |
| *F32H2.9* | *tba-6* | -2.369 | down | *C29E4.17* |  | 2.435 | up |
| *Y75B8A.38* | *sls-2.8* | -2.371 | down | *F09E5.4* | *srg-69* | 2.435 | up |
| *C25D7.14* |  | -2.372 | down | *Y26D4A.21* |  | 2.435 | up |
| *R10E11.9* |  | -2.373 | down | *Y73C8C.10* |  | 2.435 | up |
| *C35C5.11* |  | -2.373 | down | *F40D4.12* |  | 2.435 | up |
| *W04G3.11* |  | -2.376 | down | *K04A8.4* | *twk-10* | 2.435 | up |
| *T06D8.2* |  | -2.380 | down | *T24C4.3* |  | 2.435 | up |
| *T21D9.1* | *col-164* | -2.380 | down | *ZK6.6* |  | 2.435 | up |
| *ZC239.21* |  | -2.380 | down | *R10F2.4* |  | 2.435 | up |
| *ZK381.3* | *nhr-249* | -2.386 | down | *C12D8.12* | *str-182* | 2.435 | up |
| *T21E8.1* | *pgp-6* | -2.387 | down | *W08F4.2* | *fbxb-34* | 2.435 | up |
| *T19A5.5* | *nhr-219* | -2.387 | down | *K05F6.2* | *fbxb-50* | 2.435 | up |
| *ZK337.6* | *nspd-11* | -2.388 | down | *T20G5.15* |  | 2.435 | up |
| *D1086.9* |  | -2.388 | down | *C49H3.1* | *gcy-8* | 2.435 | up |
| *Y52B11A.13* |  | -2.390 | down | *F48G7.13* |  | 2.435 | up |
| *B0462.1* |  | -2.391 | down | *F49H6.3* |  | 2.435 | up |
| *F10G2.9* | *nhr-263* | -2.392 | down | *F09C6.13* |  | 2.434 | up |
| *C17G10.10* |  | -2.392 | down | *K08D12.6* |  | 2.434 | up |
| *T26H10.2* |  | -2.393 | down | *F32D8.1* |  | 2.434 | up |
| *R07E5.6* |  | -2.395 | down | *B0222.4* | *tag-38* | 2.434 | up |
| *C51E3.2* | *srsx-27* | -2.398 | down | *Y62E10A.4* | *srsx-25* | 2.433 | up |
| *C07A12.18* |  | -2.398 | down | *F44A2.7* |  | 2.431 | up |
| *EEED8.6* | *ccpp-6* | -2.399 | down | *C09G9.8* |  | 2.430 | up |
| *T20H9.5* | *fbxa-68* | -2.399 | down | *C06C3.3* |  | 2.428 | up |
| *M02H5.7* | *nhr-123* | -2.401 | down | *T20G5.5* | *epac-1* | 2.428 | up |
| *T07D3.6* |  | -2.402 | down | *F09C6.11* |  | 2.428 | up |
| *K07A12.6* | *hot-5* | -2.404 | down | *K10G6.4* |  | 2.427 | up |
| *F11A5.5* |  | -2.404 | down | *C18D11.7* |  | 2.427 | up |
| *Y48B6A.7* | *ace-4* | -2.404 | down | *F09E10.6* |  | 2.426 | up |
| *AC7.3* |  | -2.404 | down | *F49E11.3* | *amt-2* | 2.423 | up |
| *F15E6.10* |  | -2.405 | down | *F07C3.8* | *str-94* | 2.422 | up |
| *Y69A2AR.23* |  | -2.410 | down | *R09H3.3* |  | 2.422 | up |
| *F47B8.4* |  | -2.418 | down | *F38C2.5* | *ccch-2* | 2.422 | up |
| *Y116A8B.4* |  | -2.419 | down | *F47B10.6* |  | 2.421 | up |
| *T04C12.8* |  | -2.419 | down | *Y45F10D.16* |  | 2.421 | up |
| *R07B7.14* | *nhr-207* | -2.420 | down | *T13C2.2* |  | 2.421 | up |
| *K12D9.1* |  | -2.423 | down | *Y116A8B.5* |  | 2.420 | up |
| *Y45F10B.3* |  | -2.425 | down | *VY10G11R.1* |  | 2.420 | up |
| *Y75B8A.49* |  | -2.426 | down | *F31F4.17* |  | 2.418 | up |
| *T20D4.7* |  | -2.428 | down | *T12B3.2* |  | 2.418 | up |
| *Y27F2A.8* |  | -2.429 | down | *F36F12.6* | *clec-208* | 2.418 | up |
| *T26H5.11* |  | -2.430 | down | *ZK1225.1* |  | 2.418 | up |
| *ZC132.4* |  | -2.432 | down | *C05C9.1* |  | 2.418 | up |
| *H22K11.3* |  | -2.432 | down | *Y57A10C.10* |  | 2.416 | up |
| *F47B10.8* |  | -2.434 | down | *T10C6.9* |  | 2.412 | up |
| *ZC334.9* | *ins-28* | -2.435 | down | *K08H2.9* |  | 2.409 | up |
| *K09E9.4* |  | -2.436 | down | *ZK617.1* | *unc-22* | 2.408 | up |
| *K08F4.10* | *msp-37* | -2.437 | down | *K06A1.1* | *aptf-1* | 2.407 | up |
| *R05G6.9* |  | -2.439 | down | *F53B1.3* |  | 2.406 | up |
| *C47A10.12* |  | -2.439 | down | *Y55D5A.5* | *daf-2* | 2.405 | up |
| *ZK757.1* |  | -2.445 | down | *F52E10.5* | *ifa-3* | 2.403 | up |
| *Y40H7A.9* |  | -2.445 | down | *W10G11.16* | *lgc-45* | 2.403 | up |
| *C38D4.8* | *arl-6* | -2.445 | down | *R07C3.6* | *fbxc-28* | 2.402 | up |
| *F46A8.5* |  | -2.447 | down | *T03G11.10* |  | 2.401 | up |
| *R04A9.6* |  | -2.447 | down | *F31C3.8* | *rrn-1.2* | 2.398 | up |
| *ZK285.2* |  | -2.449 | down | *C04E6.4* |  | 2.396 | up |
| *W04E12.9* |  | -2.451 | down | *T05G5.2* | *hlh-4* | 2.395 | up |
| *B0513.6* |  | -2.453 | down | *Y38E10A.26* | *nspe-2* | 2.393 | up |
| *T24A6.4* | *sri-21* | -2.454 | down | *F10G2.6* | *srx-92* | 2.393 | up |
| *F27E5.5* |  | -2.456 | down | *C08E8.3* |  | 2.390 | up |
| *W06H8.4* |  | -2.459 | down | *K06A4.2* |  | 2.388 | up |
| *C17H11.5* |  | -2.462 | down | *F21F8.5* |  | 2.386 | up |
| *F13C5.3* |  | -2.464 | down | *K07C6.3* | *cyp-35B2* | 2.386 | up |
| *C24H11.3* | *tbx-38* | -2.465 | down | *M02G9.1* |  | 2.386 | up |
| *F09C6.12* |  | -2.471 | down | *Y40A1A.1* |  | 2.384 | up |
| *F26D11.10* | *inx-4* | -2.472 | down | *T01G6.3* | *str-196* | 2.383 | up |
| *H12D21.6* |  | -2.473 | down | *T24C12.4* |  | 2.381 | up |
| *ZC395.2* | *clk-1* | -2.477 | down | *F18F11.3* | *cdh-8* | 2.378 | up |
| *F15G9.6* |  | -2.479 | down | *F48G7.9* |  | 2.378 | up |
| *Y71H2AL.2* |  | -2.484 | down | *F31C3.7* | *rrn-1.1* | 2.376 | up |
| *T02B11.7* | *nas-32* | -2.484 | down | *C44C10.3* |  | 2.368 | up |
| *K08E4.5* | *bath-37* | -2.485 | down | *T26C12.2* |  | 2.367 | up |
| *B0563.9* |  | -2.485 | down | *R07C3.1* | *clec-43* | 2.361 | up |
| *T24F1.7* |  | -2.485 | down | *R09E10.2* |  | 2.360 | up |
| *Y48B6A.4* | *eat-2* | -2.489 | down | *T08H4.2* |  | 2.360 | up |
| *F59A3.13* |  | -2.490 | down | *D1009.5* | *dylt-2* | 2.357 | up |
| *R06B9.2* | *arrd-12* | -2.493 | down | *C04G6.10* |  | 2.356 | up |
| *K06C4.1* |  | -2.493 | down | *W03G9.6* | *paf-1* | 2.351 | up |
| *M116.1* |  | -2.494 | down | *ZK328.6* |  | 2.351 | up |
| *F02C9.3* | *tat-6* | -2.495 | down | *C32B5.11* | *fbxc-41* | 2.350 | up |
| *Y54H5A.5* |  | -2.497 | down | *C09D1.1* | *unc-89* | 2.345 | up |
| *K11G12.2* | *acr-2* | -2.504 | down | *BE10.1* |  | 2.344 | up |
| *F26D10.12* | *clec-196* | -2.506 | down | *F42A6.4* | *cyp-25A5* | 2.343 | up |
| *F49H12.3* |  | -2.510 | down | *C05E4.13* | *str-262* | 2.341 | up |
| *C33C12.5* | *fbxb-38* | -2.510 | down | *ZK54.3* |  | 2.340 | up |
| *F02D8.3* | *xbx-1* | -2.510 | down | *C48B6.4* |  | 2.338 | up |
| *Y57G11C.499* |  | -2.510 | down | *T25B6.7* | *snf-12* | 2.338 | up |
| *T06E6.15* |  | -2.510 | down | *F37A4.3* |  | 2.337 | up |
| *F40F9.11* |  | -2.510 | down | *T08G2.2* |  | 2.334 | up |
| *F55C9.14* |  | -2.510 | down | *Y22D7AL.16* |  | 2.329 | up |
| *R09D1.3* |  | -2.510 | down | *C45G3.4* |  | 2.327 | up |
| *T11A5.5* | *sdz-31* | -2.510 | down | *F47B10.3* |  | 2.327 | up |
| *K03B4.8* |  | -2.510 | down | *C06G4.5* | *npr-17* | 2.324 | up |
| *Y40C7B.3* |  | -2.510 | down | *K06C4.3* | *his-21* | 2.323 | up |
| *C25E10.1* | *nhr-30* | -2.510 | down | *F37B1.3* | *gst-14* | 2.322 | up |
| *F57H12.4* | *frpr-10* | -2.510 | down | *C08F11.14* |  | 2.321 | up |
| *F10E9.10* |  | -2.510 | down | *ZK185.3* |  | 2.321 | up |
| *W09C5.3* |  | -2.510 | down | *F21C10.9* |  | 2.321 | up |
| *Y73B3A.22* | *fbxa-222* | -2.510 | down | *T02G5.3* |  | 2.321 | up |
| *B0261.9* |  | -2.510 | down | *C06C3.6* | *srh-48* | 2.321 | up |
| *F09C6.2* | *fbxa-44* | -2.510 | down | *ZK1055.4* |  | 2.320 | up |
| *K12B6.4* |  | -2.510 | down | *T01B10.1* | *grd-4* | 2.319 | up |
| *M110.2* | *twk-3* | -2.510 | down | *C04F5.3* | *unc-46* | 2.319 | up |
| *F47B8.9* | *srm-6* | -2.510 | down | *F46E10.18* |  | 2.319 | up |
| *Y102A5C.38* |  | -2.510 | down | *T22C1.2* | *glb-26* | 2.318 | up |
| *C16D6.1* |  | -2.510 | down | *T21G5.2* |  | 2.318 | up |
| *T05A8.5* |  | -2.510 | down | *F38B6.6* |  | 2.317 | up |
| *T28C6.10* |  | -2.510 | down | *F53H2.2* | *cnc-7* | 2.315 | up |
| *Y39B6A.23* |  | -2.511 | down | *Y48A6B.1* | *dmsr-5* | 2.315 | up |
| *C30B5.7* |  | -2.516 | down | *Y49E10.17* | *fbxa-218* | 2.315 | up |
| *T01B10.5* |  | -2.521 | down | *K11H12.6* |  | 2.314 | up |
| *R13A1.10* |  | -2.523 | down | *F36G9.3* |  | 2.313 | up |
| *T06E6.3* | *fbxa-199* | -2.523 | down | *C29E6.1* | *let-653* | 2.311 | up |
| *ZC239.5* |  | -2.526 | down | *T20F5.8* |  | 2.310 | up |
| *T23F11.11* |  | -2.527 | down | *F55C5.9* | *srh-16* | 2.309 | up |
| *F47B8.13* |  | -2.529 | down | *F31A3.3* |  | 2.306 | up |
| *C45E5.4* |  | -2.529 | down | *C32B5.16* | *sdz-4* | 2.305 | up |
| *R04B3.1* |  | -2.530 | down | *ZK1225.5* |  | 2.302 | up |
| *Y57A10B.2* |  | -2.533 | down | *H36L18.1* |  | 2.302 | up |
| *K07A1.7* | *cri-1* | -2.534 | down | *R193.2* |  | 2.300 | up |
| *C18G1.6* |  | -2.535 | down | *AC7.1* | *tag-49* | 2.300 | up |
| *F36F2.5* | *tax-2* | -2.538 | down | *T05A7.3* |  | 2.299 | up |
| *F07H5.6* |  | -2.541 | down | *F26A1.2* | *fkh-5* | 2.299 | up |
| *B0213.10* | *cyp-34A5* | -2.541 | down | *Y39B6A.25* |  | 2.298 | up |
| *F21A9.1* |  | -2.542 | down | *T07C4.7* | *mev-1* | 2.298 | up |
| *F08F3.12* |  | -2.544 | down | *F02H6.1* |  | 2.297 | up |
| *W04G3.12* |  | -2.553 | down | *AC8.13* |  | 2.296 | up |
| *D1081.4* |  | -2.554 | down | *F28A10.7* |  | 2.295 | up |
| *F28A10.3* |  | -2.558 | down | *D2063.2* | *oac-12* | 2.295 | up |
| *C40C9.4* |  | -2.559 | down | *B0212.6* |  | 2.294 | up |
| *C07D10.3* | *sre-3* | -2.565 | down | *F57A10.2* |  | 2.294 | up |
| *T21D12.7* |  | -2.565 | down | *M03E7.3* |  | 2.292 | up |
| *C04G6.7* |  | -2.566 | down | *C06E1.4* | *glr-1* | 2.292 | up |
| *Y53F4B.20* |  | -2.566 | down | *Y61A9LA.4* |  | 2.291 | up |
| *Y62H9A.10* |  | -2.567 | down | *F46F5.9* |  | 2.291 | up |
| *C14B9.13* |  | -2.570 | down | *F08F1.9* |  | 2.291 | up |
| *Y37H9A.2* |  | -2.571 | down | *T22A3.12* |  | 2.288 | up |
| *C26B9.2* |  | -2.573 | down | *F36H1.5* | *hrg-4* | 2.287 | up |
| *C44B12.3* |  | -2.579 | down | *R08A2.1* |  | 2.287 | up |
| *F02D8.2* | *grd-12* | -2.581 | down | *T27B1.2* | *ztf-19* | 2.286 | up |
| *T24C2.1* | *ram-5* | -2.581 | down | *T05D4.3* |  | 2.285 | up |
| *B0403.10* |  | -2.582 | down | *F10D2.9* | *fat-7* | 2.283 | up |
| *K09H11.6* |  | -2.583 | down | *F13D2.4* |  | 2.283 | up |
| *F25H2.15* |  | -2.586 | down | *Y51B9A.3* |  | 2.278 | up |
| *T02G6.10* |  | -2.588 | down | *C08A9.1* | *sod-3* | 2.274 | up |
| *E02C12.11* |  | -2.591 | down | *C31H1.5* |  | 2.273 | up |
| *Y87G2A.12* |  | -2.592 | down | *F54B11.8* |  | 2.268 | up |
| *F58A6.2* |  | -2.593 | down | *H16D19.4* |  | 2.267 | up |
| *F59H6.7* | *cya-2* | -2.593 | down | *C12D8.16* | *fipr-7* | 2.266 | up |
| *C54C8.4* |  | -2.594 | down | *R13A1.4* | *unc-8* | 2.266 | up |
| *B0412.2* | *daf-7* | -2.597 | down | *M117.5* | *fbxb-4* | 2.265 | up |
| *F53G12.9* |  | -2.598 | down | *R02F2.6* |  | 2.265 | up |
| *F26F2.1* |  | -2.600 | down | *ZK353.3* |  | 2.265 | up |
| *ZK666.1* |  | -2.601 | down | *F54H12.8* |  | 2.265 | up |
| *ZC455.15* |  | -2.613 | down | *F13E9.5* |  | 2.264 | up |
| *C34E10.7* | *cnd-1* | -2.617 | down | *F59H6.5* |  | 2.264 | up |
| *Y39G8B.10* |  | -2.619 | down | *F54E7.6* |  | 2.263 | up |
| *C04C3.9* |  | -2.619 | down | *F38H4.4* |  | 2.262 | up |
| *ZK930.6* |  | -2.620 | down | *C43F9.9* | *lgc-43* | 2.261 | up |
| *T28A11.6* |  | -2.622 | down | *ZC477.10* |  | 2.260 | up |
| *R01H10.6* | *bbs-5* | -2.623 | down | *K10D11.3* |  | 2.258 | up |
| *Y80D3A.10* | *nlp-42* | -2.625 | down | *T10B10.7* | *ocr-3* | 2.258 | up |
| *F35E12.11* |  | -2.625 | down | *Y50E8A.9* | *scrm-7* | 2.258 | up |
| *Y70C5C.2* | *clec-9* | -2.629 | down | *F33A8.10* |  | 2.256 | up |
| *Y105C5B.7* |  | -2.632 | down | *C34D4.1* |  | 2.256 | up |
| *F14F9.6* |  | -2.638 | down | *M110.7* |  | 2.255 | up |
| *T27E4.15* |  | -2.642 | down | *F22B8.1* | *srj-29* | 2.254 | up |
| *F36G3.3* |  | -2.656 | down | *F49F1.7* |  | 2.252 | up |
| *R10H1.2* | *srab-14* | -2.658 | down | *T20F10.5* |  | 2.251 | up |
| *Y71A12B.3* |  | -2.658 | down | *ZK262.8* |  | 2.250 | up |
| *E04F6.16* | *mrpr-1* | -2.658 | down | *B0416.11* |  | 2.250 | up |
| *B0331.2* |  | -2.658 | down | *T01G5.8* |  | 2.250 | up |
| *T27E4.12* |  | -2.659 | down | *K01D12.2* |  | 2.249 | up |
| *F22E5.6* |  | -2.659 | down | *C53B4.8* | *mltn-12* | 2.249 | up |
| *Y47D7A.15* |  | -2.662 | down | *T09B4.3* |  | 2.247 | up |
| *F35C5.10* | *nspb-11* | -2.663 | down | *F07C3.3* |  | 2.246 | up |
| *Y41C4A.6* |  | -2.670 | down | *C38D9.2* |  | 2.244 | up |
| *M117.8* |  | -2.670 | down | *T08G5.18* |  | 2.243 | up |
| *T22C8.1* |  | -2.672 | down | *K02D3.2* |  | 2.242 | up |
| *W04H10.4* | *clec-118* | -2.672 | down | *ZC21.8* |  | 2.241 | up |
| *F02E11.4* |  | -2.679 | down | *Y67D8B.5* |  | 2.240 | up |
| *W01C8.1* |  | -2.681 | down | *Y69E1A.3* |  | 2.239 | up |
| *C50H11.17* |  | -2.685 | down | *Y106G6D.3* |  | 2.236 | up |
| *K04D7.6* |  | -2.686 | down | *ZK353.2* |  | 2.235 | up |
| *C41G7.7* |  | -2.688 | down | *ZK131.8* | *his-14* | 2.233 | up |
| *C32H11.8* |  | -2.695 | down | *C28D4.4* |  | 2.232 | up |
| *F40H7.12* |  | -2.696 | down | *Y18D10A.10* | *clec-104* | 2.231 | up |
| *F53B6.8* | *fipr-26* | -2.699 | down | *Y9C9A.6* | *str-168* | 2.229 | up |
| *Y8G1A.1* | *math-46* | -2.699 | down | *C46G7.51* |  | 2.228 | up |
| *Y38H6C.9* |  | -2.700 | down | *T12A7.7* |  | 2.227 | up |
| *R05G6.5* |  | -2.701 | down | *F07B7.4* | *his-52* | 2.222 | up |
| *Y60A3A.23* |  | -2.707 | down | *C06C6.7* |  | 2.222 | up |
| *F33E2.4* |  | -2.707 | down | *ZK1053.6* |  | 2.222 | up |
| *T02H6.9* |  | -2.713 | down | *R08A2.2* |  | 2.218 | up |
| *C02B4.8* |  | -2.721 | down | *F58A4.12* |  | 2.216 | up |
| *Y116A8C.43* |  | -2.721 | down | *F31D4.6* | *try-4* | 2.215 | up |
| *C52B9.5* | *srv-6* | -2.723 | down | *F31C3.15* |  | 2.215 | up |
| *F53A2.11* |  | -2.737 | down | *Y80D3A.8* |  | 2.215 | up |
| *T28A11.11* | *gst-23* | -2.740 | down | *F53F8.6* |  | 2.211 | up |
| *F35C5.11* |  | -2.740 | down | *Y47H9C.1* |  | 2.211 | up |
| *ZK84.4* |  | -2.744 | down | *Y39D8B.1* | *mltn-7* | 2.210 | up |
| *T15B7.10* |  | -2.745 | down | *T08G5.11* |  | 2.210 | up |
| *Y105C5A.1269* |  | -2.746 | down | *F09F9.4* |  | 2.210 | up |
| *ZK105.4* | *srsx-40* | -2.748 | down | *C15C7.4* |  | 2.209 | up |
| *F48B9.5* | *npax-2* | -2.751 | down | *F41F3.1* |  | 2.209 | up |
| *H09G03.1* |  | -2.751 | down | *F22E10.4* | *pgp-15* | 2.209 | up |
| *C09G12.1* | *ceh-53* | -2.754 | down | *F36H12.16* |  | 2.209 | up |
| *Y43F8C.11* |  | -2.756 | down | *F01D4.8* |  | 2.209 | up |
| *ZK550.7* |  | -2.756 | down | *M28.7* | *nphp-1* | 2.209 | up |
| *F46B6.11* | *sru-39* | -2.756 | down | *C43D7.2* | *fbxb-65* | 2.209 | up |
| *F58G6.2* | *srm-3* | -2.756 | down | *M03B6.1* |  | 2.209 | up |
| *F36H12.17* |  | -2.756 | down | *F30H5.5* |  | 2.209 | up |
| *R05H10.7* |  | -2.756 | down | *C05C8.10* |  | 2.206 | up |
| *E02H9.1* |  | -2.756 | down | *D2092.7* | *tsp-19* | 2.206 | up |
| *F56A12.1* | *unc-39* | -2.756 | down | *T21E12.2* |  | 2.205 | up |
| *K05G3.1* |  | -2.763 | down | *Y54G11A.10* | *lin-7* | 2.204 | up |
| *K07C5.10* |  | -2.763 | down | *F35H10.11* | *his-29* | 2.203 | up |
| *Y5H2A.2* | *nhr-275* | -2.764 | down | *C07E3.10* |  | 2.202 | up |
| *K08H2.2* |  | -2.766 | down | *F42A10.8* | *nas-28* | 2.201 | up |
| *C24A8.5* |  | -2.776 | down | *F21H7.10* |  | 2.200 | up |
| *F53E10.5* |  | -2.791 | down | *K10C8.2* | *frpr-15* | 2.197 | up |
| *W04B5.6* |  | -2.794 | down | *Y106G6G.2* |  | 2.196 | up |
| *C03C10.7* |  | -2.799 | down | *F11C1.3* | *scav-4* | 2.195 | up |
| *F21E9.4* | *ins-39* | -2.805 | down | *C47F8.8* | *nhr-81* | 2.193 | up |
| *K04F1.1* |  | -2.805 | down | *C06C3.7* |  | 2.189 | up |
| *ZK686.6* |  | -2.814 | down | *C55C3.1* |  | 2.189 | up |
| *F14E5.8* |  | -2.816 | down | *F58A6.6* | *srb-16* | 2.188 | up |
| *Y73F8A.22* |  | -2.819 | down | *F40E10.5* |  | 2.186 | up |
| *R186.2* | *srd-35* | -2.829 | down | *F13H10.5* |  | 2.185 | up |
| *T04C12.14* |  | -2.829 | down | *H12D21.12* | *nspa-2* | 2.184 | up |
| *C15A7.1* | *lgc-23* | -2.830 | down | *R07H5.10* | *zip-6* | 2.184 | up |
| *R02C2.5* | *str-121* | -2.832 | down | *T24A6.16* |  | 2.184 | up |
| *T24D8.4* | *nlp-23* | -2.832 | down | *Y73F8A.20* |  | 2.184 | up |
| *C36B7.7* | *hen-1* | -2.834 | down | *W09B7.2* |  | 2.184 | up |
| *C04C3.6* |  | -2.838 | down | *E02H9.6* |  | 2.183 | up |
| *F46E10.3* |  | -2.848 | down | *F55A3.5* |  | 2.182 | up |
| *K06G5.3* |  | -2.850 | down | *R05D8.10* | *dhs-15* | 2.182 | up |
| *F07E5.7* |  | -2.852 | down | *F13E9.12* |  | 2.182 | up |
| *W01B6.7* | *col-2* | -2.853 | down | *H12D21.14* | *nspa-6* | 2.181 | up |
| *Y54G2A.44* |  | -2.854 | down | *T09B9.3* |  | 2.180 | up |
| *Y37F4.5* |  | -2.857 | down | *F49B2.6* |  | 2.175 | up |
| *D1054.18* |  | -2.862 | down | *R12E2.18* |  | 2.174 | up |
| *Y37A1B.12* | *tor-1* | -2.864 | down | *Y49F6C.1* | *bath-8* | 2.172 | up |
| *Y37E3.22* |  | -2.864 | down | *R03D7.8* |  | 2.170 | up |
| *F21A3.2* |  | -2.864 | down | *Y71D11A.5* | *lgc-46* | 2.170 | up |
| *B0462.4* |  | -2.864 | down | *Y52D5A.2* |  | 2.169 | up |
| *C32B5.13* |  | -2.864 | down | *C16H3.1* |  | 2.168 | up |
| *C43D7.10* |  | -2.864 | down | *Y48G1BM.6* |  | 2.167 | up |
| *C45H4.10* | *srbc-24* | -2.864 | down | *F31D4.7* | *twk-35* | 2.166 | up |
| *C49C3.3* |  | -2.864 | down | *ZK75.1* | *ins-4* | 2.165 | up |
| *F21D9.1* |  | -2.864 | down | *F28H7.8* |  | 2.165 | up |
| *F25E5.7* |  | -2.864 | down | *T25E12.16* |  | 2.164 | up |
| *F55B12.9* | *srx-129* | -2.864 | down | *Y73F8A.32* |  | 2.164 | up |
| *R12B2.7* |  | -2.864 | down | *ZK328.7* |  | 2.164 | up |
| *T08B6.1* |  | -2.864 | down | *F09D12.2* |  | 2.162 | up |
| *T10H4.9* | *srx-51* | -2.864 | down | *F21A9.2* |  | 2.159 | up |
| *Y57G7A.9* | *glb-31* | -2.864 | down | *F22F1.2* |  | 2.158 | up |
| *Y76B12C.1* | *cng-2* | -2.864 | down | *C18E9.8* |  | 2.157 | up |
| *ZK262.10* | *srj-26* | -2.864 | down | *C07A9.4* | *ncx-6* | 2.157 | up |
| *F54E2.2* |  | -2.864 | down | *C04E6.12* |  | 2.156 | up |
| *C29F7.11* |  | -2.864 | down | *C08F11.6* |  | 2.156 | up |
| *C49C3.13* | *clec-198* | -2.864 | down | *R04B5.11* |  | 2.156 | up |
| *F57F4.2* |  | -2.864 | down | *Y41E3.18* |  | 2.153 | up |
| *K09H11.11* |  | -2.864 | down | *ZK185.4* |  | 2.153 | up |
| *T05B11.6* | *srr-10* | -2.864 | down | *Y57G11C.50* |  | 2.153 | up |
| *T11F9.22* |  | -2.864 | down | *W01G7.2* |  | 2.153 | up |
| *Y49F6C.7* |  | -2.864 | down | *F49F1.14* |  | 2.153 | up |
| *Y51H7BR.8* |  | -2.864 | down | *F49H6.13* |  | 2.150 | up |
| *Y54E5B.5* |  | -2.864 | down | *F53G12.3* | *duox-2* | 2.150 | up |
| *F39E9.12* | *btb-18* | -2.864 | down | *C07A9.6* | *ugt-60* | 2.146 | up |
| *F28A10.2* |  | -2.864 | down | *ZK643.1* | *arrd-15* | 2.145 | up |
| *F19B10.7* | *srx-97* | -2.864 | down | *F49E12.4* | *ubc-24* | 2.143 | up |
| *C08H9.4* |  | -2.864 | down | *R09B5.1* | *fbxa-195* | 2.142 | up |
| *C38C6.4* | *sre-13* | -2.864 | down | *C44C10.5* |  | 2.142 | up |
| *Y26G10.6* |  | -2.864 | down | *F39E9.22* |  | 2.141 | up |
| *T11F9.5* | *nas-21* | -2.864 | down | *K02F6.4* |  | 2.140 | up |
| *F58G1.9* |  | -2.864 | down | *Y6G8.1* | *srz-45* | 2.140 | up |
| *F54F7.8* |  | -2.872 | down | *T07A5.1* |  | 2.139 | up |
| *F45D3.1* |  | -2.878 | down | *C05E7.4* | *frpr-2* | 2.136 | up |
| *C44H4.1* | *lron-1* | -2.879 | down | *K09A9.5* | *gas-1* | 2.135 | up |
| *T17A3.6* | *fbxb-83* | -2.879 | down | *T16A1.2* |  | 2.133 | up |
| *F41D3.11* |  | -2.879 | down | *F48F7.7* | *arrd-24* | 2.133 | up |
| *C05B5.10* |  | -2.879 | down | *R04D3.10* | *srxa-8* | 2.133 | up |
| *K01A2.9* |  | -2.879 | down | *C26D10.7* |  | 2.132 | up |
| *F17A9.7* |  | -2.882 | down | *K09A11.4* | *cyp-14A3* | 2.132 | up |
| *Y57G11B.8* |  | -2.883 | down | *ZK180.8* |  | 2.132 | up |
| *Y17G9A.4* |  | -2.884 | down | *K03B4.5* | *srx-77* | 2.130 | up |
| *Y39E4A.1* |  | -2.887 | down | *F36G9.12* | *oac-20* | 2.129 | up |
| *K07E3.9* |  | -2.889 | down | *Y106G6H.9* |  | 2.129 | up |
| *C02E7.2* | *srh-21* | -2.893 | down | *F59D6.2* |  | 2.127 | up |
| *C07G3.3* | *str-227* | -2.896 | down | *C31H2.3* |  | 2.126 | up |
| *B0244.15* |  | -2.899 | down | *F58F12.3* |  | 2.126 | up |
| *F38H4.2* | *fbxb-3* | -2.906 | down | *Y110A2AM.4* |  | 2.126 | up |
| *F45G2.1* | *nas-1* | -2.906 | down | *C27D9.2* |  | 2.126 | up |
| *C03C10.9* |  | -2.908 | down | *F36H12.5* |  | 2.125 | up |
| *B0205.5* |  | -2.910 | down | *F31D4.8* |  | 2.125 | up |
| *B0281.6* |  | -2.920 | down | *T20B6.2* |  | 2.124 | up |
| *F11F1.7* | *ttr-52* | -2.928 | down | *K01A11.1* |  | 2.124 | up |
| *Y102A5B.2* | *clec-35* | -2.929 | down | *W09D6.7* | *sls-2.14* | 2.123 | up |
| *M04C7.1* | *gpa-15* | -2.931 | down | *C24G7.2* | *acd-1* | 2.122 | up |
| *F46A8.6* |  | -2.931 | down | *T22D1.18* |  | 2.121 | up |
| *B0284.6* |  | -2.931 | down | *H42K12.1* | *pdk-1* | 2.121 | up |
| *C01G12.6* | *nspb-10* | -2.933 | down | *C07A9.11* | *ncx-7* | 2.120 | up |
| *E02A10.2* | *grl-23* | -2.945 | down | *F52H3.6* |  | 2.120 | up |
| *F45F2.6* | *otpl-6* | -2.947 | down | *F10D11.1* | *sod-2* | 2.119 | up |
| *F39B2.9* |  | -2.952 | down | *K01B6.3* |  | 2.118 | up |
| *F36D4.6* |  | -2.973 | down | *C24H12.1* |  | 2.117 | up |
| *C01B7.7* |  | -2.978 | down | *W10G11.4* |  | 2.116 | up |
| *F26D2.3* |  | -2.988 | down | *Y55F3C.3* | *kvs-5* | 2.115 | up |
| *Y50E8A.10* |  | -2.992 | down | *ZK112.5* |  | 2.115 | up |
| *F59F5.5* |  | -2.995 | down | *H19M22.2* | *let-805* | 2.114 | up |
| *C37A5.6* |  | -2.997 | down | *Y39B6A.41* |  | 2.114 | up |
| *K03H6.4* |  | -2.997 | down | *F37C12.14* |  | 2.114 | up |
| *F40C5.3* | *grl-27* | -3.002 | down | *C18C4.7* |  | 2.113 | up |
| *C45E5.3* |  | -3.004 | down | *R07E5.5* |  | 2.112 | up |
| *K05F6.7* | *fbxb-54* | -3.005 | down | *T21E3.2* |  | 2.110 | up |
| *C13A2.4* |  | -3.011 | down | *F23H11.7* |  | 2.109 | up |
| *F40G9.6* |  | -3.016 | down | *Y53C12B.5* | *mab-3* | 2.109 | up |
| *C54D10.9* |  | -3.040 | down | *K09E10.2* | *oac-58* | 2.109 | up |
| *Y53C10A.15* |  | -3.054 | down | *K09B11.4* |  | 2.108 | up |
| *T19H12.5* | *srd-32* | -3.064 | down | *F48E3.8* |  | 2.105 | up |
| *F18C5.1* | *sra-27* | -3.067 | down | *ZK783.6* |  | 2.103 | up |
| *F09E10.10* |  | -3.083 | down | *F23H12.7* |  | 2.103 | up |
| *K08D12.7* |  | -3.087 | down | *F22D6.11* | *gly-18* | 2.102 | up |
| *T03E6.9* |  | -3.101 | down | *C06E1.7* |  | 2.102 | up |
| *Y46C8AR.3* | *clec-77* | -3.103 | down | *C06G4.6* |  | 2.101 | up |
| *C14A6.8* |  | -3.103 | down | *B0024.15* |  | 2.099 | up |
| *W03F9.3* |  | -3.106 | down | *C33F10.8* |  | 2.097 | up |
| *F17E9.13* | *his-33* | -3.114 | down | *C27B7.6* |  | 2.096 | up |
| *F23D12.1* |  | -3.115 | down | *K05D4.4* | *cyp-33D1* | 2.096 | up |
| *F36G9.7* |  | -3.116 | down | *F56A6.5* |  | 2.096 | up |
| *F55A4.9* | *rncs-1* | -3.117 | down | *C33G8.3* |  | 2.096 | up |
| *C50H2.4* |  | -3.118 | down | *F20D6.2* |  | 2.096 | up |
| *B0205.14* |  | -3.120 | down | *M01B2.1* | *kin-30* | 2.096 | up |
| *W01B6.8* |  | -3.128 | down | *C30H6.3* | *clec-201* | 2.096 | up |
| *F32G8.3* |  | -3.148 | down | *C47E8.9* |  | 2.096 | up |
| *Y59E9AL.1* | *thn-6* | -3.154 | down | *T23B3.4* | *ckr-1* | 2.096 | up |
| *Y110A2AL.10* |  | -3.163 | down | *R01E6.5* |  | 2.096 | up |
| *F38B6.7* |  | -3.187 | down | *R01H2.8* |  | 2.096 | up |
| *Y48A6C.6* |  | -3.197 | down | *R04B5.8* | *srd-49* | 2.096 | up |
| *C34C6.1* | *srg-15* | -3.198 | down | *C42D4.4* | *str-44* | 2.096 | up |
| *W09C3.3* |  | -3.202 | down | *Y116A8C.5* |  | 2.096 | up |
| *C28H8.8* |  | -3.204 | down | *Y43D4A.6* |  | 2.096 | up |
| *C17E7.13* |  | -3.206 | down | *R07B7.12* |  | 2.096 | up |
| *ZK809.10* |  | -3.215 | down | *Y80D3A.3* | *ceh-51* | 2.096 | up |
| *F56H6.2* |  | -3.216 | down | *C07B5.6* |  | 2.096 | up |
| *F15A4.3* | *sre-37* | -3.223 | down | *R08H2.8* |  | 2.096 | up |
| *W09C5.12* |  | -3.224 | down | *T14G12.4* | *fkh-2* | 2.096 | up |
| *F53A9.4* | *jbts-14* | -3.224 | down | *K05F6.9* | *fbxb-46* | 2.096 | up |
| *B0304.9* | *sra-36* | -3.228 | down | *T23B3.3* | *rmd-5* | 2.096 | up |
| *T20G5.6* | *unc-47* | -3.236 | down | *F09C12.2* |  | 2.096 | up |
| *T04A8.1* | *srg-10* | -3.236 | down | *T08E11.6* | *fbxb-10* | 2.096 | up |
| *C09G12.6* | *srz-70* | -3.247 | down | *K03B4.6* |  | 2.096 | up |
| *C18H7.8* | *srt-59* | -3.247 | down | *F22B8.5* | *str-7* | 2.096 | up |
| *T28F3.6* | *ifta-2* | -3.247 | down | *F23H12.9* | *fipr-2* | 2.096 | up |
| *F36F2.8* |  | -3.247 | down | *F32H2.8* |  | 2.095 | up |
| *F52B11.8* |  | -3.247 | down | *C06E7.6* | *spe-27* | 2.093 | up |
| *C01B9.5* |  | -3.247 | down | *F19F10.5* | *ets-7* | 2.092 | up |
| *K09H11.4* |  | -3.247 | down | *W03B1.7* | *oac-51* | 2.092 | up |
| *C39E6.2* |  | -3.247 | down | *T04C12.23* |  | 2.091 | up |
| *F26F2.10* |  | -3.247 | down | *R102.10* |  | 2.091 | up |
| *ZK1053.1* |  | -3.247 | down | *T01G5.2* | *ugt-30* | 2.090 | up |
| *C43F9.4* |  | -3.247 | down | *F20A1.7* | *twk-11* | 2.089 | up |
| *T26E3.8* |  | -3.247 | down | *T07H6.4* |  | 2.089 | up |
| *F36H5.11* | *fbxb-12* | -3.247 | down | *C14C11.4* |  | 2.089 | up |
| *E04A4.1* | *fbxb-78* | -3.247 | down | *F09C6.3* |  | 2.089 | up |
| *F48D6.3* | *hlh-13* | -3.247 | down | *C47F8.2* | *nhr-165* | 2.088 | up |
| *F59A7.4* | *hil-6* | -3.247 | down | *Y47G6A.17* |  | 2.088 | up |
| *F49B2.1* | *fbxb-8* | -3.247 | down | *Y39B6A.7* |  | 2.088 | up |
| *F36H5.4* |  | -3.261 | down | *AH10.3* |  | 2.085 | up |
| *Y73B6A.2* |  | -3.263 | down | *Y45F10B.59* |  | 2.085 | up |
| *F59E11.6* |  | -3.266 | down | *C17D12.5* |  | 2.084 | up |
| *Y37A1B.18* |  | -3.277 | down | *C23H5.11* |  | 2.084 | up |
| *F09C6.5* | *srp-9* | -3.299 | down | *C29G2.5* | *srt-58* | 2.083 | up |
| *W01D2.6* |  | -3.301 | down | *C08H9.1* |  | 2.083 | up |
| *T13F3.7* |  | -3.305 | down | *C42C1.2* |  | 2.082 | up |
| *Y18H1A.14* |  | -3.306 | down | *R03D7.3* |  | 2.082 | up |
| *K10D6.1* | *lgc-49* | -3.307 | down | *M05B5.1* |  | 2.082 | up |
| *T20G5.12* |  | -3.311 | down | *C09E7.6* |  | 2.082 | up |
| *T21E12.3* |  | -3.325 | down | *T19H12.1* | *ugt-9* | 2.082 | up |
| *K06A4.10* |  | -3.330 | down | *T22B11.4* |  | 2.081 | up |
| *E04F6.10* |  | -3.334 | down | *T01C8.3* |  | 2.081 | up |
| *F11H8.8* |  | -3.343 | down | *R09D1.9* |  | 2.079 | up |
| *R07B1.7* |  | -3.349 | down | *T04H1.6* | *lrx-1* | 2.078 | up |
| *F29D10.3* |  | -3.352 | down | *T23F11.2* |  | 2.076 | up |
| *F54B11.2* | *col-44* | -3.370 | down | *F53H1.2* |  | 2.074 | up |
| *F02A9.7* |  | -3.389 | down | *ZK1290.7* |  | 2.074 | up |
| *C42C1.6* |  | -3.417 | down | *F49F1.1* |  | 2.073 | up |
| *C18A11.4* |  | -3.421 | down | *W01F3.3* | *mlt-11* | 2.073 | up |
| *ZC196.2* |  | -3.432 | down | *F52H2.15* |  | 2.072 | up |
| *F39E9.18* |  | -3.435 | down | *Y38F2AL.7* |  | 2.071 | up |
| *B0250.8* |  | -3.441 | down | *F14E12.3* |  | 2.071 | up |
| *C40A11.2* |  | -3.457 | down | *C48C5.3* | *aexr-3* | 2.070 | up |
| *C37A5.5* |  | -3.469 | down | *C39E6.4* | *mls-2* | 2.070 | up |
| *W04H10.1* |  | -3.476 | down | *C54G7.3* | *lgx-1* | 2.068 | up |
| *Y105C5A.17* |  | -3.486 | down | *W01B6.4* |  | 2.067 | up |
| *C01B12.5* |  | -3.494 | down | *ZC455.1* |  | 2.067 | up |
| *K04C2.6* | *med-2* | -3.505 | down | *Y18H1A.10* |  | 2.066 | up |
| *C06C6.6* |  | -3.505 | down | *AC8.5* |  | 2.065 | up |
| *Y17G9A.3* |  | -3.505 | down | *ZK1025.9* | *nhr-113* | 2.064 | up |
| *Y77E11A.16* |  | -3.518 | down | *W03F9.4* |  | 2.064 | up |
| *Y71H2AM.25* |  | -3.523 | down | *R01H2.7* |  | 2.064 | up |
| *F37C4.7* |  | -3.527 | down | *F54E2.3* | *ketn-1* | 2.064 | up |
| *F45F2.4* | *his-7* | -3.531 | down | *Y73B6BL.36* |  | 2.063 | up |
| *F58E1.3* | *fbxb-26* | -3.536 | down | *T28B8.3* |  | 2.062 | up |
| *M02A10.2* | *irk-2* | -3.538 | down | *T12A2.16* | *hot-4* | 2.062 | up |
| *F09C12.6* |  | -3.550 | down | *R02F2.5* |  | 2.061 | up |
| *R102.9* | *glb-21* | -3.552 | down | *Y69H2.1* |  | 2.061 | up |
| *F36D3.3* | *srr-3* | -3.568 | down | *T23G11.10* |  | 2.061 | up |
| *C27H5.6* |  | -3.593 | down | *C01G10.16* |  | 2.060 | up |
| *Y39H10A.1* |  | -3.598 | down | *T18H9.5* | *inx-10* | 2.060 | up |
| *B0310.6* |  | -3.602 | down | *Y57G7A.5* |  | 2.059 | up |
| *F26F2.8* |  | -3.602 | down | *R07E3.3* | *cut-5* | 2.059 | up |
| *K09E9.5* |  | -3.616 | down | *Y37D8A.8* |  | 2.059 | up |
| *Y43F8B.25* |  | -3.622 | down | *C53D6.10* |  | 2.059 | up |
| *K02A2.2* | *srd-55* | -3.631 | down | *T10B9.1* | *cyp-13A4* | 2.059 | up |
| *ZK1025.8* |  | -3.641 | down | *B0285.11* |  | 2.058 | up |
| *Y119C1B.12* |  | -3.680 | down | *T05E8.2* | *hil-8* | 2.058 | up |
| *F29F11.5* | *ceh-22* | -3.722 | down | *T17H7.1* |  | 2.058 | up |
| *K09F6.11* |  | -3.734 | down | *Y53F4B.51* |  | 2.058 | up |
| *T08E11.8* |  | -3.738 | down | *K07C11.10* |  | 2.057 | up |
| *F26E4.16* |  | -3.742 | down | *B0240.2* | *spe-42* | 2.057 | up |
| *EGAP2.2* |  | -3.744 | down | *T15B7.4* | *col-142* | 2.056 | up |
| *F47E1.5* |  | -3.744 | down | *Y41C4A.18* |  | 2.055 | up |
| *ZC513.2* |  | -3.752 | down | *R13H9.2* | *msp-57* | 2.053 | up |
| *ZK666.10* | *sri-8* | -3.773 | down | *C49F5.1* | *sams-1* | 2.053 | up |
| *37C4.2* | *oac-22* | -3.773 | down | *ZC190.1* | *cln-3.3* | 2.053 | up |
| *B0034.5* |  | -3.774 | down | *D1086.17* |  | 2.052 | up |
| *Y45F10B.8* |  | -3.823 | down | *C12D8.10* | *akt-1* | 2.052 | up |
| *EGAP4.1* |  | -3.837 | down | *C17C3.9* |  | 2.051 | up |
| *C08F8.15* |  | -3.858 | down | *T05C3.2* |  | 2.051 | up |
| *Y45F3A.8* |  | -3.900 | down | *T25C12.1* | *lin-14* | 2.051 | up |
| *F58B4.2* |  | -3.932 | down | *K06A1.2* |  | 2.050 | up |
| *B0331.1* | *cyp-29A4* | -3.936 | down | *ZK945.8* |  | 2.049 | up |
| *W09B6.5* |  | -3.940 | down | *K08F4.8* | *msp-38* | 2.049 | up |
| *R08E5.4* |  | -3.952 | down | *C01H6.4* |  | 2.048 | up |
| *F46F5.10* |  | -3.959 | down | *W03G1.1* | *glt-7* | 2.048 | up |
| *C44B11.1* |  | -3.968 | down | *R01H2.2* |  | 2.047 | up |
| *D1065.4* | *srh-210* | -3.970 | down | *Y39C12A.5* | *sre-16* | 2.047 | up |
| *T13C5.3* |  | -3.984 | down | *R09H10.7* |  | 2.044 | up |
| *T08E11.5* | *fbxc-19* | -3.984 | down | *F25F2.2* | *cdh-4* | 2.042 | up |
| *Y26D4A.2* | *hpo-2* | -3.984 | down | *F26C11.1* |  | 2.041 | up |
| *F10C2.7* |  | -3.984 | down | *C53D6.3* | *acc-2* | 2.041 | up |
| *ZK899.6* |  | -3.984 | down | *K06C4.6* | *mod-1* | 2.040 | up |
| *C32B5.15* |  | -3.984 | down | *F14F9.3* |  | 2.039 | up |
| *F08D12.10* | *sdz-9* | -3.984 | down | *B0365.7* | *dhc-3* | 2.037 | up |
| *F13A7.13* | *sre-36* | -3.992 | down | *F38B2.3* |  | 2.036 | up |
| *H25P06.5* |  | -4.033 | down | *Y6D1A.3* |  | 2.036 | up |
| *T21B4.12* | *srx-118* | -4.036 | down | *F40G9.15* |  | 2.035 | up |
| *F43G9.11* | *ces-1* | -4.061 | down | *Y53F4B.38* |  | 2.034 | up |
| *F57E7.1* |  | -4.084 | down | *F14H3.10* | *cyp-35D1* | 2.033 | up |
| *Y56A3A.10* | *fbxb-22* | -4.089 | down | *R155.5* |  | 2.033 | up |
| *C34B4.5* |  | -4.104 | down | *F44F1.4* |  | 2.032 | up |
| *F10D2.5* | *ugt-40* | -4.111 | down | *K09C8.5* | *pxn-2* | 2.032 | up |
| *H08J19.1* |  | -4.121 | down | *BE10.5* |  | 2.031 | up |
| *F48D6.2* | *srv-5* | -4.134 | down | *C45E5.1* |  | 2.029 | up |
| *C14A6.7* |  | -4.140 | down | *T20F10.4* |  | 2.028 | up |
| *F42E11.3* |  | -4.143 | down | *K08E5.3* | *mua-3* | 2.028 | up |
| *T05A8.2* |  | -4.146 | down | *K07C5.13* |  | 2.028 | up |
| *T22F3.5* | *srh-213* | -4.146 | down | *C29F5.3* |  | 2.023 | up |
| *C06B8.10* | *srh-218* | -4.146 | down | *C05G5.6* | *let-7* | 2.023 | up |
| *C14C6.4* | *nhr-155* | -4.146 | down | *R05G9.5* |  | 2.022 | up |
| *C17E7.5* | *nhr-157* | -4.146 | down | *K08E7.6* |  | 2.022 | up |
| *C43D7.11* |  | -4.146 | down | *H12D21.13* | *nspa-4* | 2.021 | up |
| *F14B6.5* | *oac-16* | -4.146 | down | *K12D12.4* |  | 2.020 | up |
| *F19B10.11* |  | -4.146 | down | *C03F11.1* | *kcnl-3* | 2.019 | up |
| *T15D6.10* |  | -4.146 | down | *C03F11.2* |  | 2.019 | up |
| *Y106G6G.8* |  | -4.146 | down | *F55F1.5* |  | 2.019 | up |
| *Y49F6B.5* | *sdz-32* | -4.146 | down | *C09G9.4* | *tag-19* | 2.019 | up |
| *Y67A10A.14* |  | -4.146 | down | *ZK218.22* | *sls-1.12* | 2.019 | up |
| *Y37H2A.10* |  | -4.146 | down | *T05B4.14* |  | 2.018 | up |
| *C04E12.10* |  | -4.146 | down | *R07B1.3* | *scav-5* | 2.017 | up |
| *Y66D12A.1* |  | -4.146 | down | *F11E6.5* | *elo-2* | 2.016 | up |
| *F44E5.2* |  | -4.146 | down | *C49C3.20* |  | 2.016 | up |
| *T10H9.6* | *str-180* | -4.146 | down | *Y39G10AR.16* |  | 2.015 | up |
| *Y46G5A.37* |  | -4.146 | down | *H02I12.3* | *tag-89* | 2.015 | up |
| *Y32G9A.2* |  | -4.147 | down | *K04D7.4* |  | 2.014 | up |
| *E03H4.4* |  | -4.168 | down | *K07F5.1* | *msp-81* | 2.012 | up |
| *T23B12.5* |  | -4.176 | down | *F33D11.6* |  | 2.009 | up |
| *T04B8.2* |  | -4.207 | down | *Y44A6B.4* | *ttr-13* | 2.009 | up |
| *F16A11.5* |  | -4.224 | down | *T28C12.2* |  | 2.009 | up |
| *C25B8.7* | *aexr-2* | -4.230 | down | *C35E7.2* |  | 2.007 | up |
| *Y71A12B.2* |  | -4.259 | down | *T19D12.5* |  | 2.007 | up |
| *ZC334.1* | *ins-26* | -4.263 | down | *C09G1.3* |  | 2.006 | up |
| *C10A4.7* |  | -4.306 | down | *T28A11.4* |  | 2.005 | up |
| *B0285.12* |  | -4.332 | down | *Y73B3A.t1* |  | 2.005 | up |
| *W04D2.8* |  | -4.353 | down | *K04E7.2* | *pept-1* | 2.005 | up |
| *Y37E11B.9* |  | -4.365 | down | *K02D10.3* |  | 2.004 | up |
| *T20D4.15* |  | -4.374 | down | *F41E6.12* |  | 2.0032 | up |
| *Y51H4A.28* | *col-136* | -4.383 | down | *C01G10.1* |  | 2.003 | up |
| *F21G4.3* |  | -4.404 | down | *C09B9.3* |  | 2.001 | up |
| *ZK643.9* |  | -4.426 | down | *T12B3.1* |  | 2.001 | up |
| *F40B1.2* | *bath-18* | -4.426 | down | *C01B9.1* |  | 2.000 | up |
| *R102.1* |  | -4.437 | down | *T01B6.1* |  | 2.000 | up |
| *F02D10.1* | *col-183* | -4.445 | down | *ZK1251.11* | *ins-8* | 2.000 | up |
| *F41E6.10* | *fip-4* | -4.455 | down |  |  |  |  |
| *Y113G7A.22* |  | -4.492 | down |  |  |  |  |
| *C09F5.3* |  | -4.502 | down |  |  |  |  |
| *C31A11.4* | *srbc-82* | -4.507 | down |  |  |  |  |
| *C45H4.13* |  | -4.531 | down |  |  |  |  |
| *Y106G6G.3* | *dlc-6* | -4.604 | down |  |  |  |  |
| *F56H1.10* |  | -4.608 | down |  |  |  |  |
| *K11G12.3* | *smf-2* | -4.623 | down |  |  |  |  |
| *F32D1.8* |  | -4.636 | down |  |  |  |  |
| *Y60A3A.31* |  | -4.647 | down |  |  |  |  |
| *ZK697.14* |  | -4.673 | down |  |  |  |  |
| *B0554.7* |  | -4.681 | down |  |  |  |  |
| *Y17D7C.2* |  | -4.694 | down |  |  |  |  |
| *B0365.2* |  | -4.699 | down |  |  |  |  |
| *C37C3.11* |  | -4.700 | down |  |  |  |  |
| *F28B12.6* |  | -4.721 | down |  |  |  |  |
| *F34D6.1* |  | -4.721 | down |  |  |  |  |
| *F26D2.15* |  | -4.740 | down |  |  |  |  |
| *F23H11.6* |  | -4.753 | down |  |  |  |  |
| *C54C8.8* |  | -4.832 | down |  |  |  |  |
| *C27F2.6* |  | -4.945 | down |  |  |  |  |
| *B0454.10* | *sri-30* | -4.973 | down |  |  |  |  |
| *T24A6.21* | *grl-32* | -5.008 | down |  |  |  |  |
| *T05B4.1* | *lgc-1* | -5.083 | down |  |  |  |  |
| *T06H11.11* |  | -5.212 | down |  |  |  |  |
| *R08B4.4* |  | -5.314 | down |  |  |  |  |
| *T18D3.5* |  | -5.355 | down |  |  |  |  |
| *Y9C9A.14* | *srz-27* | -5.365 | down |  |  |  |  |
| *K11E4.3* |  | -5.418 | down |  |  |  |  |
| *C01A2.1* |  | -5.429 | down |  |  |  |  |
| *T25E12.12* | *fbxa-127* | -5.429 | down |  |  |  |  |
| *F48E3.7* | *lgc-11* | -5.429 | down |  |  |  |  |
| *H34P18.1* | *dmsr-12* | -5.429 | down |  |  |  |  |
| *F28G4.3* |  | -5.429 | down |  |  |  |  |
| *Y41G9A.2* |  | -5.429 | down |  |  |  |  |
| *Y55F3AM.2* | *srx-12* | -5.429 | down |  |  |  |  |
| *M01D1.9* | *fbxb-40* | -5.429 | down |  |  |  |  |
| *W02D7.9* |  | -5.432 | down |  |  |  |  |
| *F42G2.7* |  | -5.458 | down |  |  |  |  |
| *B0286.6* | *try-9* | -5.795 | down |  |  |  |  |
| *F58E2.11* |  | -5.831 | down |  |  |  |  |
| *Y6B3B.7* |  | -5.837 | down |  |  |  |  |
| *F52C6.14* |  | -5.933 | down |  |  |  |  |
| *ZC204.9* | *fbxb-20* | -6.195 | down |  |  |  |  |
| *M01D1.8* | *fbxb-41* | -6.195 | down |  |  |  |  |
| *F09F9.5* |  | -6.586 | down |  |  |  |  |
| *K09C8.8* |  | -6.618 | down |  |  |  |  |
| *Y55F3C.10* |  | -6.649 | down |  |  |  |  |
| *C16C4.5* | *math-15* | -6.685 | down |  |  |  |  |
| *R02F11.9* |  | -6.711 | down |  |  |  |  |
| *F41C6.6* |  | -6.711 | down |  |  |  |  |
| *C53D6.5* |  | -6.711 | down |  |  |  |  |
| *F01G12.18* |  | -6.76 | down |  |  |  |  |
| *Y43F4A.4* |  | -7.352 | down |  |  |  |  |
| *Y47G6A.30* |  | -7.456 | down |  |  |  |  |
| *C49D10.8* | *oac-11* | -7.591 | down |  |  |  |  |
| *T11F9.10* |  | -7.596 | down |  |  |  |  |
| *Y37E11AL.12* |  | -7.723 | down |  |  |  |  |
| *F26F12.2* |  | -7.877 | down |  |  |  |  |
| *C49G7.8* |  | -7.916 | down |  |  |  |  |
| *F41C3.6* |  | -7.959 | down |  |  |  |  |
| *C14H10.4* | *str-74* | -7.994 | down |  |  |  |  |
| *K03H6.1* |  | -7.994 | down |  |  |  |  |
| *F14D7.7* |  | -8.049 | down |  |  |  |  |
| *T26H2.10* |  | -8.599 | down |  |  |  |  |
| *M01E5.1* |  | -9.289 | down |  |  |  |  |
| *K02D7.6* | *grl-26* | -10.438 | down |  |  |  |  |
| *C54C8.9* | *nlp-39* | -10.558 | down |  |  |  |  |
| *T20B6.1* |  | -10.558 | down |  |  |  |  |

Note:

FC, fold changes.

The blue color indicates the genes associated with the control of oxidative stress, the red color indicates the genes associated with the control of intestinal development, and the purple color indicates the genes encoding insulin signaling pathway.

**Table S2 | Gene ontology terms with gene counts more than 10 based on down-regulated mRNAs in MWCNTs exposed nematodes.**

| No. | GO term | GO ID | Counts | GO type |
| --- | --- | --- | --- | --- |
| 1 | Metabolic process | GO:0008152 | 292 | Biological process |
| 2 | Cellular process | GO:0009987 | 240 | Biological process |
| 3 | Catalytic activity | GO:0003824 | 186 | Molecular function |
| 4 | Binding | GO:0005488 | 159 | Molecular function |
| 5 | Biological regulation | GO:0065007 | 131 | Biological process |
| 6 | Localization | GO:0051179 | 118 | Biological process |
| 7 | Receptor activity | GO:0004872 | 117 | Molecular function |
| 8 | Cell part | GO:0044464 | 106 | Cellular component |
| 9 | Transporter activity | GO:0005215 | 77 | Molecular function |
| 10 | Multicellular organismal process | GO:0032501 | 65 | Biological process |
| 11 | Response to stimulus | GO:0050896 | 63 | Biological process |
| 12 | Organelle | GO:0043226 | 58 | Cellular component |
| 13 | Developmental process | GO:0032502 | 53 | Biological process |
| 14 | Nucleic acid binding transcription factor activity | GO:0001071 | 52 | Molecular function |
| 15 | Cellular component organization or biogenesis | GO:0071840 | 45 | Biological process |
| 16 | Membrane | GO:0016020 | 43 | Cellular component |
| 17 | Structural molecule activity | GO:0005198 | 39 | Molecular function |
| 18 | Macromolecular complex | GO:0032991 | 32 | Cellular component |
| 19 | Immune system process | GO:0002376 | 26 | Biological process |
| 20 | Enzyme regulator activity | GO:0030234 | 22 | Molecular function |
| 21 | Extracellular region | GO:0005576 | 21 | Cellular component |
| 22 | Biological adhesion | GO:0022610 | 14 | Biological process |
| 23 | Extracellular matrix | GO:0031012 | 12 | Cellular component |
| 24 | Apoptotic process | GO:0006915 | 4 | Biological process |
| 25 | Translation regulator activity | GO:0045182 | 4 | Molecular function |
| 26 | Reproduction | GO:0000003 | 3 | Biological process |

Note:

All the listed gene ontology terms were statistically significant (*P* < 0.05).

**Table S3 | Gene ontology terms with gene counts more than 10 based on up-regulated mRNAs in MWCNTs exposed nematodes.**

| No. | GO term | GO ID | Counts | GO type |
| --- | --- | --- | --- | --- |
| 1 | Metabolic process | GO:0008152 | 312 | Biological process |
| 2 | Cellular process | GO:0009987 | 239 | Biological process |
| 3 | Catalytic activity | GO:0003824 | 200 | Molecular function |
| 4 | Binding | GO:0005488 | 168 | Molecular function |
| 5 | Biological regulation | GO:0065007 | 150 | Biological process |
| 6 | Localization | GO:0051179 | 116 | Biological process |
| 7 | Cell part | GO:0044464 | 103 | Cellular component |
| 8 | Receptor activity | GO:0004872 | 96 | Molecular function |
| 9 | Transporter activity | GO:0005215 | 82 | Molecular function |
| 10 | Multicellular organismal process | GO:0032501 | 79 | Biological process |
| 11 | Organelle | GO:0043226 | 61 | Cellular component |
| 12 | Nnucleic acid binding transcription factor activity | GO:0001071 | 59 | Molecular function |
| 13 | Response to stimulus | GO:0050896 | 53 | Biological process |
| 14 | Developmental process | GO:0032502 | 51 | Biological process |
| 15 | Membrane | GO:0016020 | 46 | Cellular component |
| 16 | Cellular component organization or biogenesis | GO:0071840 | 36 | Biological process |
| 17 | Enzyme regulator activity | GO:0030234 | 31 | Molecular function |
| 18 | Immune system process | GO:0002376 | 30 | Biological process |
| 19 | Structural molecule activity | GO:0005198 | 29 | Molecular function |
| 20 | Macromolecular complex | GO:0032991 | 25 | Cellular component |
| 21 | Biological adhesion | GO:0022610 | 13 | Biological process |
| 22 | Extracellular region | GO:0005576 | 9 | Cellular component |
| 23 | Reproduction | GO:0000003 | 9 | Biological process |
| 24 | Translation regulator activity | GO:0045182 | 6 | Molecular function |
| 25 | Apoptotic process | GO:0006915 | 5 | Biological process |
| 26 | Extracellular matrix | GO:0031012 | 4 | Cellular component |

Note:

All the listed gene ontology terms were statistically significant (*P* < 0.05).

**Table S4 | Signaling pathways with the gene number more than 2 based on down-regulated mRNAs in MWCNTs exposed nematodes.**

| No. | Pathway | Counts |
| --- | --- | --- |
| 1 | 5HT type receptor mediated signaling pathway | 7 |
| 2 | Alzheimer disease-amyloid secretase pathway | 4 |
| 3 | Alzheimer disease-presenilin pathway | 3 |
| 4 | Angiogenesis | 3 |
| 5 | Apoptosis signaling pathway | 2 |
| 6 | Axon guidance mediated by netrin | 2 |
| 7 | Cadherin signaling pathway | 3 |
| 8 | Cytoskeletal regulation by Rho GTPase | 4 |
| 9 | Axon guidance mediated by Slit/Robo | 2 |
| 10 | BMP signaling pathway | 2 |
| 11 | Cell cycle | 2 |
| 12 | EGF receptor signaling pathway | 5 |
| 13 | FAS signaling pathway | 2 |
| 14 | FGF signaling pathway | 6 |
| 15 | DPP signaling pathway | 2 |
| 16 | Gonadotropin releasing hormone receptor pathway | 1 |
| 17 | Huntington disease | 6 |
| 18 | Endothelin signaling pathway | 3 |
| 19 | Inflammation mediated signaling pathway | 4 |
| 20 | Insulin/IGF pathway | 2 |
| 21 | Integrin signalling pathway | 6 |
| 22 | Metabotropic glutamate receptor pathway | 2 |
| 23 | Interferon-gamma signaling pathway | 2 |
| 24 | Muscarinic acetylcholine receptor signaling pathway | 5 |
| 25 | Nicotinic acetylcholine receptor signaling pathway | 12 |
| 26 | Oxytocin receptor mediated signaling pathway | 2 |
| 27 | p53 pathway | 4 |
| 28 | Oxidative stress response | 2 |
| 29 | Parkinson disease | 11 |
| 30 | PDGF signaling pathway | 2 |
| 31 | Ras pathway | 3 |
| 32 | TGF-beta signaling pathway | 7 |
| 33 | hormone receptor signaling pathway | 2 |
| 34 | Toll receptor signaling pathway | 3 |
| 35 | Transcription regulation by bZIP transcription factor | 3 |
| 36 | Ubiquitin proteasome pathway | 6 |
| 37 | VEGF signaling pathway | 3 |
| 38 | Insulin signaling pathway | 2 |

Note:

All the listed signaling pathways were statistically significant (*P* < 0.05).

**Table S5 | Signaling pathways with the gene number more than 2 based on up-regulated mRNAs in MWCNTs exposed nematodes.**

| No. | Pathway | Counts |
| --- | --- | --- |
| 1 | 5HT type receptor mediated signaling pathway | 1 |
| 2 | Alzheimer disease-presenilin pathway | 4 |
| 3 | Asparagine and aspartate biosynthesis | 3 |
| 4 | Axon guidance mediated by netrin | 1 |
| 5 | Cadherin signaling pathway | 3 |
| 6 | Cytoskeletal regulation by Rho GTPase | 2 |
| 7 | EGF receptor signaling pathway | 2 |
| 8 | FAS signaling pathway | 1 |
| 9 | FGF signaling pathway | 3 |
| 10 | Gamma-aminobutyric acid synthesis | 2 |
| 11 | Gonadotropin releasing hormone receptor pathway | 2 |
| 12 | Heme biosynthesis | 2 |
| 13 | Huntington disease | 2 |
| 14 | Inflammation mediated by chemokine and cytokine signaling pathway | 3 |
| 15 | MAP kinase cascade | 1 |
| 16 | Insulin/IGF pathway | 1 |
| 17 | Integrin signalling pathway | 3 |
| 18 | Ionotropic glutamate receptor pathway | 2 |
| 19 | Metabotropic glutamate receptor group III pathway | 2 |
| 20 | Nicotinic acetylcholine receptor signaling pathway | 2 |
| 21 | Oxytocin receptor mediated signaling pathway | 1 |
| 22 | p53 pathway | 4 |
| 23 | Parkinson disease | 2 |
| 24 | PDGF signaling pathway | 4 |
| 25 | PI3 kinase pathway | 3 |
| 26 | Ras pathway | 1 |
| 27 | TCA cycle | 2 |
| 28 | TGF-beta signaling pathway | 1 |
| 29 | Transcription regulation by bZIP transcription factor | 2 |
| 30 | Ubiquitin proteasome pathway | 2 |
| 31 | VEGF signaling pathway | 1 |
| 32 | Wnt signaling pathway | 5 |
| 33 | 5HT type receptor mediated signaling pathway | 1 |
| 34 | Alzheimer disease-presenilin pathway | 4 |
| 35 | Asparagine and aspartate biosynthesis | 3 |
| 36 | Insulin signaling pathway | 4 |

Note:

All the listed signaling pathways were statistically significant (*P* < 0.05).

**Table S6 | microRNAs-mRNAs networks involved in the control of MWCNTs toxicity.**

| microRNA | Target gene(s) |
| --- | --- |
| *mir-228* | *sek-1*, *gnrr-8*, *ztf-19*, *C45E5.4*,*T14E8.4* |
| *mir-249* | *lin-14*, *isp-1*, *ZK816.1*, *amt-2*, *mls-2*, *hmit-1.2* |
| *mir-47* | *T22B11.4*, *T24C12.4*, *inx-10* |
| *mir-355* | *ZK757.1*, *Y116A8C.43*, *pmk-1*, *dig-1*, *C01G10.16*, *R09H10.6*, *F59F5.5*, *mod-1*, *T22C8.1*, *T25C8.1*, *daf-2*, *folt-3*, *sek-1*, *T05A1.5* |
| *mir-45* | *ceh-45*, *cdh-8*, *unc-22*, *F59D12.2*, *ckr-1*, *C55C3.1*, *ketn-1*, *lin-14*, *ces-1*, *unc-55* |
| *mir-2210* | *F26D10.11* |
| *mir-57* | *mod-1*, *R05G9R.1* |
| *mir-1018* | *lin-14*, *sek-1*, *T19D12.7*, *mab-3*, *dig-1* |
| *mir-360* | *lin-14*, *jnk-1* |
| *lin-4* | *Y43F4A.4*, *B0334.13*, *lin-14* |
| *mir-64* | *lin-14*, *ajm-1*, *mls-2*, *D1054.18*, *ztf-19*, *pag-3*, *srv-5*, *nas-13*, *bbs-8*, *ceh-30*, *inx-3* |
| *mir-2209* | *tag-89*, *ceh-9*, *ztf-19*, *hrg-4*, *T04F8.9*, *srr-10*, *F48E3.8*, *F32G8.3*, *pag-3*, *B0462.4*, *ZK816.1*, *glb-8*, *Y73F8A.32*, *T24C12.4*, *T01B10.5*, *lag-2*, *C30B5.7*, *M01H9.2*, *C06G4.6*, *ceh-30*, *Y47D3B.3*, *T27E4.5*, *twk-23*, *ZK682.7*, *F47B10.8*, *K01A12.3*, *T01C3.11*, *ckr-1*, *folt-3*, *C06G1.2*, *ace-4*, *tbx-7* |
| *mir-793* | *rnt-1*, *cri-1*, *lin-14* |
| *mir-1830* | *C04E6.4*, *lin-14*, *acr-2* |
| *mir-2210* | *F26D10.11* |
| *mir-83* | *let-805*, *cdh-8*, *C45E5.4*, *cdh-4* |
| *mir-789* | *ZC455.1*, *F28C6.10*, *W09G10.3*, *R02E12.5*, *Y48G8AR.2, F09F7.1* |
| *mir-4806* | *unc-46* |

Note: The blue color indicates the genes associated with the control of oxidative stress, the red color indicates the genes associated with the control of intestinal development, and the purple color indicates the genes encoding insulin signaling pathway.

**Table S7 | Primers used for quantitative real-time polymerase chain reaction (PCR).**

| Gene | Forward primer | Reverse primer |
| --- | --- | --- |
| *tba-1* | TCAACACTGCCATCGCCGCC | TCCAAGCGAGACCAGGCTTCAG |
| *daf-16* | CAAGCGTGGAACTGTCGT | AGCCGAACACGAACAACA |
| *daf-2* | ATGTGGCGTGAGAATGAA | AGCCGAACACGAACAACA |
| *daf-18* | ATCATCATCCGCCGAGTC | ACCGTTGAGTCCTCCATC |
| *pdk-1* | TTCAGAGCCGTCAACCAG | GCTCACTTGCTCGGCTTT |
| *akt-1* | GGACAACCGTTTCCTGAG | GACGAACTTCTGCCGACT |
| *age-1* | ATGGAAACCGCCGAGTGT | ATTGGCAGTCGGTTCAGG |
